# Material Specificity Drives Medial Temporal Lobe Familiarity But Not Hippocampal Recollection

**DOI:** 10.1002/hipo.22683

**Published:** 2016-12-26

**Authors:** Alex Kafkas, Ellen M. Migo, Robin G. Morris, Michael D. Kopelman, Daniela Montaldi, Andrew R. Mayes

**Affiliations:** ^1^ Memory Research Unit, School of Biological Sciences, Division of Neuroscience & Experimental PsychologyUniversity of ManchesterUnited Kingdom; ^2^ Institute of Psychiatry, Psychology & NeuroscienceKing's College LondonUnited Kingdom

**Keywords:** recognition memory, parahippocampal cortex, perirhinal cortex, entorhinal cortex, amygdala

## Abstract

The specific role of the perirhinal (PRC), entorhinal (ERC) and parahippocampal cortices (PHC) in supporting familiarity‐based recognition remains unknown. An fMRI study explored whether these medial temporal lobe (MTL) structures responded in the same way or differentially to familiarity as a function of stimulus type at recognition. A secondary aim was to explore whether the hippocampus responds in the same way to equally strong familiarity and recollection and whether this is influenced by the kind of stimulus involved. Univariate and multivariate analyses revealed that familiarity responses in the PRC, ERC, PHC and the amygdala are material‐specific. Specifically, the PRC and ERC selectively responded to object familiarity, while the PHC responded to both object and scene familiarity. The amygdala only responded to familiarity memory for faces. The hippocampus did not respond to stimulus familiarity for any of the three types of stimuli, but it did respond to recollection for all three types of stimuli. This was true even when recollection was contrasted to equally accurate familiarity. Overall, the findings suggest that the role of the MTL neocortices and the amygdala in familiarity‐based recognition depends on the kind of stimulus in memory, whereas the role of the hippocampus in recollection is independent of the type of cuing stimulus. © 2016 The Authors Hippocampus Published by Wiley Periodicals, Inc.

**Abbreviations**
ERC: entorhinal cortex
GPC: Gaussian Process Classification
MTL: medial temporal lobe
MVPA: multi‐voxel pattern analysis
PHC: parahippocampal cortice
PRC: perirhinal cortex

## INTRODUCTION

Recognizing a stimulus, such as a face, an object or a scene (e.g., a landscape), as something that has been encountered before involves a pivotal human ability called recognition memory. This can be supported by a feeling of memory that the stimulus has been encountered before and/or by the recall of specific contextual details about a previous encounter with it. These two kinds of memory are called familiarity and recollection, respectively, and have been proposed to depend on partially distinct psychological and neural encoding, storage and retrieval processes (Mandler, 1980; Jacoby, 1991; Aggleton and Brown, 1999; Yonelinas, 2002; Eichenbaum et al., 2007; Montaldi and Mayes, 2010). Familiarity memory has long been thought to rely on the Medial Temporal Lobe (MTL) and extra‐MTL cortical areas. Nevertheless, the specific role of regions such as the perirhinal (PRC), entorhinal (ERC), and parahippocampal (PHC) cortices is still uncertain. In the present study, we used fMRI to explore the interaction between stimulus content and the kind of memory engaged by testing recognition. The primary focus was to determine to what extent the patterns of MTL response found when familiarity for different types of stimuli occurs are shared or distinct. This constitutes a pivotal question relating to the degree of functional specialization for familiarity decisions within the MTL cortices. It should be noted here that we use the term MTL cortices to describe the neocortical PRC, ERC and PHC, but not the archicortical hippocampus or the subcortical amygdala.

### Familiarity‐Based Recognition and Material‐Specificity

Material‐specific effects have so far been studied almost exclusively for recollection or recollection‐related processes, such as in associative tasks requiring the retrieval of different kinds of source information (Duarte et al., 2011; Staresina et al., 2011, 2013; Hannula et al., 2013). The neural networks that support familiarity memory have been investigated less systematically (but see Montaldi et al., 2006; Kafkas and Montaldi, 2012, 2014) and the interaction between familiarity‐based recognition and kind of stimulus has received less attention. This question is particularly important in order to evaluate the role of the different neocortical MTL structures (PRC, PHC and ERC) in supporting familiarity‐based recognition.

Most models of the MTL's role in recognition memory (Aggleton and Brown, 1999; Eichenbaum et al., 2007; Montaldi and Mayes, 2010) agree that the hippocampus plays a special role in mediating recollection, while familiarity‐based recognition is mediated by the adjacent neocortical MTL structures, most prominently the PRC (Squire et al., 2007; Wixted et al., 2010). There is less agreement about the role of the PHC with some theories linking its role to recollection (Diana et al., 2007) although it has also been linked to familiarity for contextual information (Montaldi and Mayes, 2010). Similar uncertainty applies to the role of the ERC. Specifically, it is unclear whether the ERC, which receives inputs from both PHC and PRC, subserves familiarity and, if it does, for what kind of information. Although, there is some evidence linking it to familiarity‐based recognition for words (Ranganath et al., 2004; Yonelinas et al., 2007; de Vanssay‐Maigne et al., 2011; Brandt et al., 2016).

Indirect neuroanatomical evidence strongly suggests that the PRC, PHC and ERC processing their inputs in a similar way, which is distinct from how the hippocampus processes its inputs. They share similar neocortical cytoarchitectonics, which differs from the archicortical cytoarchitectonics of the hippocampus. Furthermore, the distinct inputs of the PHC and PRC from structures outside the MTL is consistent with the hypothesis that they subserve familiarity for different kinds of information (see Montaldi and Mayes, 2010). However, considering the inputs from within the MTL, particularly the reciprocal connections between PHC and PRC (Suzuki and Amaral, 1994), both structures may mediate familiarity for the same inputs but perhaps at different time points.

It is widely believed that PRC processes object‐related inputs whereas PHC processes scene, spatial or context inputs (Davachi, 2006; Eichenbaum et al., 2007). That PRC and PHC process different kinds of information has been supported by fMRI studies, in which the PRC has usually been reported to selectively process and represent high‐level object and face information, whereas the PHC has been involved in processing scene information (Epstein et al., 1999; Lee et al., 2005, 2008; Awipi and Davachi, 2008; Litman et al., 2009; Staresina et al., 2011, 2013). Nevertheless, the evidence so far is not conclusive and findings inconsistent with PRC and PHC processing respectively only object‐related versus scene/context‐related information, have also been reported (Bar and Aminoff, 2003; Buffalo et al., 2006; Diana et al., 2008; Preston et al., 2010).

For example, Preston et al. (2010), using repeated and novel face and scene stimuli in an fMRI study, found that encoding activation only in PHC selectively related to scenes, whereas activation in PRC related to subsequent memory (old/new recognition judgments) for both faces and scenes. Selective responding in the PHC for scene encoding, but a similar response profile within the PRC for faces and scenes was also reported by Dudukovic et al. (2011). On the other hand, Diana et al. (2008) found that activity within the PHC is sensitive not only to scenes but also to faces and toys, whereas activity in the PRC did not respond to any of the employed stimulus categories. These inconsistencies could be attributed to differences in experimental design in different studies. However, they may also reflect an interaction between stimulus category and the underlying memory process, such as familiarity or recollection, which participants may engage during repeated presentations of stimuli.

As noted above, all the studies that have explored material‐specificity in the MTL have to a great extent ignored whether familiarity or recollection are involved in recognition decisions or they have exclusively focused on recollection or other associative tasks. One notable exception is a recent study by Martin et al. (2013; for a neurospychological study see also Martin et al., 2011) in which familiarity‐based recognition for three types of objects (faces, buildings and chairs) was contrasted within the PRC and the PHC. In this study, a preference in the PRC for faces, but not buildings, and in the PHC for buildings but not faces was reported. However, it remains to a great extent unexplored whether the PRC and PHC have a general (i.e., non‐specific) role in supporting familiarity memory for every stimulus category (e.g., faces, objects and scenes) or whether they are parts of networks that are specialized in supporting familiarity‐based recognition for specific types of stimuli. This question is directly explored in the present study.

### The Role of the Hippocampus in Recollection

In contrast to the MTL cortices, the hippocampus has been proposed to have a general role in recollection and in associative memory (Aggleton and Brown, 1999; Eichenbaum et al., 2007; Mayes et al., 2007; Montaldi and Mayes, 2010) across different domains and stimulus categories (Davachi, 2006; Konkel and Cohen, 2009; Duarte et al., 2011; Staresina et al., 2011). Nevertheless, this proposal has been criticized and an important controversy remains as to whether the hippocampus has a selective role in recollection or supports both familiarity and recollection. Recent fMRI and neuropsychological evidence (for reviews see Eichenbaum et al., 2007; Skinner and Fernandes, 2007; Montaldi and Mayes, 2010; Migo et al., 2012; Rugg and Vilberg, 2013) linking recollection to the hippocampus, but not to familiarity, has been challenged on the grounds that these findings are subject to a methodological confound (Squire et al., 2007; Wixted et al., 2010). According to this argument, hippocampal activity and familiarity and recollection decisions systematically co‐vary with *recognition memory strength*, resulting in higher hippocampal activity for recollection, which is usually associated with stronger recognition memories. In contrast, although sometimes familiarity can support strong recognition memory, it is usually associated with weaker recognition memories that fail to noticeably affect the hippocampal activity. The construct of “memory strength” in this argument is operationally defined as recognition memory *accuracy* and reported *confidence*.

A key prediction of this argument, therefore, is that when familiarity and recollection responses are equally strong—as indicated by the response accuracy—the hippocampus should be engaged in both cases. However, recent fMRI studies provide mixed results, either supporting (Smith et al., 2011) or contradicting (Kafkas and Montaldi, 2012) this key prediction of the strength confound view. However, these studies also had a key difference in method: the category of stimuli used. An important factor, therefore, that needs to be addressed in studies of recognition memory is whether the type of information that is being encoded or retrieved has any effect on which neural systems are engaged when familiarity and recollection decisions are taken (for a discussion of this issue see also Kafkas and Montaldi, 2012).

### The Present Study

In the present study, we set out to explore MTL responses to familiarity and recollection supporting the recognition of three different types of stimuli: objects, faces and scenes. As outlined above, our primary aim was to explore whether familiarity responses for objects, faces and scenes differentially engage the neocortical MTL structures, the PRC, the ERC and the PHC. A secondary aim of the present experiment was to investigate whether hippocampal activity changes relate to recollection, but not to familiarity, even when familiarity is as strong (i.e., accurate) as recollection and whether this is consistent for object, face and scene recognition memory. In our analyses we combined univariate‐GLM and multivariate (pattern recognition) approaches. The combination of the two methods of analysis has the potential to provide complementary findings and bridge inconsistencies reported in previous studies.

## MATERIALS AND METHODS

### Participants

In total, 20 right‐handed healthy volunteers gave informed consent and participated in this experiment. All participants were native English speakers, with no self‐reported psychiatric or neurological disorders and normal or corrected‐to‐normal vision (with contact lenses). Data from three participants were excluded from further analyses; one due to excessive movement during fMRI (more than 3 mm), one due to a technical problem affecting the recording of the fMRI data and another one due to chance memory performance at retrieval. The mean age of the remaining 17 participants (12 male) was 23.28 years (SD = 3.40 years). All participants received £20 after completing the testing session. The National Research Ethics Service (North West‐GM South) approved all the procedures followed in this experiment.

### Stimulus Material

A total of 420 color stimuli (15 for practice) were used in this experiment. As the aim of the study was to explore the brain networks that support familiarity‐based and recollection‐based recognition for different stimulus types, three stimulus categories were used: outdoor scenes, objects (man‐made and natural) and faces. For each stimulus type 140 stimuli (five for practice) were presented. The scene and the object stimuli (70 man‐made and 70 natural) were royalty‐free images with transferable copyrights collected from various online databases (e.g., the BOSS object database; Brodeur et al., 2010), whereas the faces (70 male and 70 female) were selected from the Glasgow face database (Burton et al., 2010).

### Procedure and Design

**Figure 1:**
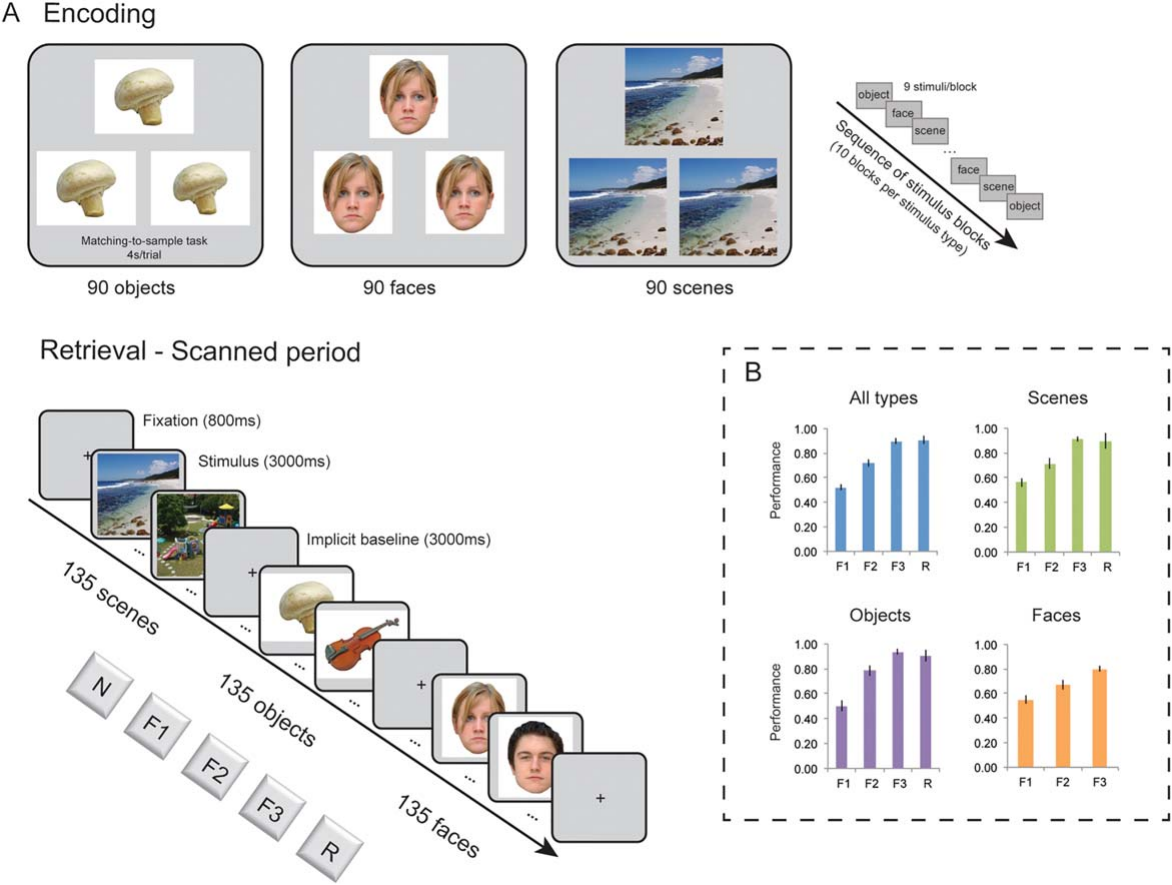
Experimental design and behavioral data. (A) Design of the fMRI experiment and sequence of trials at encoding and retrieval (scanned). Three types of stimuli (scenes, objects and faces) were studied at encoding and were later presented at retrieval (along with unstudied stimuli). Participants were asked to provide familiarity (*F*1, *F*2, *F*3), new (N) and recollection (*R*) responses for each stimulus. (B) Accuracy [Hits/(Hits + FAs)] collapsed across the three types of stimuli and separately for scenes, objects and faces. Recollection responses to faces were very rare and therefore are not reported separately. Error bars show the standard error of the mean. [Color figure can be viewed at wileyonlinelibrary.com]

Each experimental session comprised an encoding phase (before scanning) and a retrieval phase completed in the MRI scanner (Fig. 1). At encoding, participants were presented with 270 stimuli depicting outdoor scenes, objects and faces (90 stimuli per type), organized in 10 randomly alternating blocks of nine stimuli each. All three types of stimuli used a perceptual matching‐to‐sample task. This shallow encoding task (see Montaldi et al., 2006; Kafkas and Montaldi, 2012, 2014) involves taking a matching‐to‐sample decision based on a perceptual dimension of the presented stimulus. Specifically, each trial comprised image triplets of the same stimulus and participants had to decide which of the two bottom images matched the target image presented on top (see Fig. 1). In each trial, one of the two bottom images had been minimally modified from the original image according to a perceptual characteristic. For the objects and the faces the modified image was slightly smaller or bigger whereas, for the scenes, the modified image was slightly shifted horizontally (left or right) by a few mm. In each trial, the modified picture was placed randomly on the left or the right side of the screen and participants had 4s to indicate their response using two keyboard buttons (“1” for left and “0” for right). A practice block, exemplifying this task for the three types of stimuli, preceded the main encoding block. In our previous studies (Kafkas & Montaldi, 2012, 2014, 2015a; Montaldi et al., 2006) and in pilot work, this encoding task increased reliance on familiarity‐based recognition keeping the frequency of the recollection responses at lower levels—without, nevertheless, affecting recollection accuracy, which remains high. This task also ensures an adequate distribution of responses across familiarity levels, which is necessary for the parametric analyses of the brain activation data (see below).

After completing the encoding task, participants underwent the retrieval task in the scanner. On seeing each stimulus, participants were required to focus on judging quickly but accurately how familiar each stimulus felt across three levels: weakly, moderately or strongly familiar. If, however, they spontaneously recollected something about having seen the stimulus earlier, then they were required to respond with the ‘recollect’ option. Participants were required not to *try* to recollect at any time (this 'familiarity‐only' adaption of the remember/know procedure is described by Montaldi et al., 2006; Mayes et al., 2007; Kafkas and Montaldi, 2012). Participants were carefully trained beforehand so that they understood the distinction between familiarity and recollection (see Kafkas and Montaldi 2012, 2015b; Migo et al., 2012). Specifically, they were trained to make a familiarity response when they felt that they had seen a stimulus at study, but to make a recollection response when a stimulus brought to mind (albeit spontaneously) information associated with encoding it during its earlier encounter (e.g., that the stimulus was one of the first seen or it triggered a specific thought at encoding). Participants had the opportunity to ask questions about these two kinds of memory and provide examples of each of them from their own past experience.

A practice retrieval block was completed before the participants entered the scanner and another practice block was presented as they lay in the MRI scanner when the structural T1 image was being acquired, before the main retrieval task. The fMRI data were collected and analyzed for this main retrieval phase, which was divided into two functional runs. Participants were presented with 270 stimuli from the encoding task (i.e., target items at encoding) along with 135 new foils (i.e., 405 stimuli across all stimulus types). For each stimulus type, 135 stimuli per category (90 studied) were presented at retrieval while participants provided responses using the three levels of increasing familiarity (*F*1 = weak, *F*2 = moderate, *F*3 = strong familiarity), spontaneous recollection (*R*) and new (*N*) options (Fig. 1).

At retrieval, stimuli were presented in a pseudo‐random sequence of face, object and scene blocks, separated by short fixation periods (10s). Within this sequence each block was followed by a different stimulus block. Each of these blocks comprised 15 trials intermixed with three implicit baseline fixation trials (null events). In total, 81 null events were presented across all the blocks. Each trial (including the null events) lasted for 3s followed by a 1s fixation cross. Participants were instructed to provide a response for each stimulus trial within this period, using a special MR‐compatible button box. As the recognition task included five possible responses, three buttons on one hand were used for the familiarity responses (*F*1, *F*2, and *F*3) and two buttons on the other hand were used for the extra two responses (*R* and *N*). The assignment of these responses to left and right hand was counterbalanced across participants.

### fMRI Acquisition and Analyses

Scanning was conducted on a 3T Philips (Achieva) scanner. A gradient echo‐planar pulse sequence was used for the acquisition of the functional data using the blood oxygenation level dependent (BOLD) contrast. In total, 840 volumes were acquired, for each participant, across two sessions with TR = 2.5s, TE = 35ms, 2.5mm × 2.5mm × 3.5mm voxel size and 40 slices per volume covering the whole brain (positioned parallel to the AC‐PC). High‐resolution T1 images were also acquired prior to the functional run (180 slices with a voxel size of 1mm isotropic and matrix size 256 × 256). Soft pads were used to minimize head motion during scanning and earplugs were provided to reduce MRI scanner noise.

Data quality of the fMRI time‐series was examined using the *ArtRepair* software (http://cibsr.stanford.edu/tools/human-brain-project/artrepair-software.html). Fewer than 5% of the slices from two subjects were repaired using ArtRepair algorithms implementing linear interpolation of the adjacent (preceding and following) slices within the time‐series. The other 15 participants did not have any major artifacts in their functional data and no repair algorithms were applied. The EPI data from each participant were pre‐processed and analysed using SPM8 software (Statistical Parametric Mapping, Wellcome Trust Centre for Neuroimaging, http://www.fil.ion.ucl.ac.uk/spm/). The fMRI time‐series were realigned to the mean image (registration) using a six‐parameter rigid body transformation, resliced using sinc interpolation and slice‐time corrected (to the middle slice) to account for differences in slice acquisition times. Residual movement artefacts were also obtained from the ArtRepair toolbox and used as nuisance regressors in the first‐level analysis (see below). Individual T1 images were coregistered to the corresponding mean EPI image. Spatial normalization of the EPI and T1 images to the MNI template was performed using the DARTEL toolbox implemented in SPM8 (Ashburner, 2007). Finally, the spatially normalized EPI data were resliced to 3 mm isotropic and spatially smoothed using an isotropic 6mm full width half maximum (FWHM) Gaussian kernel.

### Univariate Analyses

The pre‐processed individual EPI data were further analyzed in SPM8, first at a single subject level using the general linear model (GLM analysis). Specifically, the event‐related functional data were modelled separately for each participant using a canonical hemodynamic response function (Friston et al., 1998). In this model a series of delta (stick) functions corresponding to the onset of each event were convolved with the canonical hemodynamic response. Two models, one parametric and one categorical were specified for each participant (see below) and included all response outcomes for each stimulus type (objects, faces and scenes) modeled as conditions of interest. Nuisance regressors were also modeled and included trials with no behavioral response, the six movement parameters, produced at realignment for each of the two functional runs and residual movement artefacts obtained from the ArtRepair toolbox. To remove low‐frequency noise the data were high‐pass filtered using a cut‐off of 128s.

#### Parametric analyses

Brain activity modulations by familiarity strength were investigated using the parametric model and involved three parametric analyses, exploring monotonic increases or decreases in activity across familiarity, separately for scenes, objects and faces (Büchel et al., 1998). Specifically, at the first (subject) level, familiarity hits (i.e., old stimuli reported as familiar), for each stimulus type were specified as separate conditions, while the reported strength accompanying each familiarity response was used as a covariate and was convolved with the stimulus‐specific HRF. Three parametric conditions were specified in this way; one for objects, one for scenes and one for faces, each of them comprising the four levels of familiarity strength (*F*0, *F*1, *F*2, and *F*3), with misses used as the level reflecting zero familiarity (*F*_0_). All participants had a minimum of 9 trials in each response category for each stimulus type enabling reliable parametric analyses (for the mean number of trials across the four response categories for the three types of stimuli see Supporting Information Table 1). Parametric *t* contrasts were created at the first‐level for monotonic increases or decreases in activity across familiarity strength for each stimulus type. Non‐linear quadratic effects were also modeled to capture residual variance not explained by the linear function; however, these produced no significant additional activations and are not reported separately. Finally, the parametric analyses were also conducted using three levels of strength (*F*1, *F*2, and *F*3) for each type of stimulus, but these produced very similar results to the main analyses and are not presented separately.

In the parametric analyses both common (i.e., shared) activations across all stimulus types as well as material‐specific familiarity activations for each type of stimulus were examined. Specifically, shared familiarity activity was explored by means of a conjunction analysis (Friston et al., 2005) testing for consistent parametric effects in the whole brain across scenes, objects and faces. Unique parametric activation and deactivation patterns across familiarity strength were explored for each stimulus type (scenes, objects and faces) by applying a series of exclusive masks (at *P* < 0.05) to each parametric contrast. In the case of scene familiarity, the parametric responses were exclusively masked by object and face familiarity activations. Similarly, object familiarity activations and deactivations were exclusively masked by scene and face familiarity activation and deactivation patterns. Finally, parametric responses to familiarity for faces were exclusively masked by familiarity responses to scenes and objects.

#### Familiarity versus recollection contrasts

To explore whether the hippocampus preferentially responds to recollection or to both recollection and strong familiarity (*F*3), a direct contrast between these two conditions was run for objects (*R*_objects_ > *F*3_objects_) and scenes (*R*_scenes_ > *F*3_scences_) and collapsed across the three types of stimuli (*R* > *F*3) using the categorical model. Furthermore, two conjunction analyses were conducted to assess the overlap of the hippocampal responses for scene and object stimuli, one using the *R* > *F*3 contrast and the other using the *R* > *M* contrast for the two types of stimuli. As faces produced very few *R* responses, these were not analyzed separately or in the conjunction analyses, but were included in the analysis with the collapsed *R* responses. Furthermore, *F*3 and *R* responses were also contrasted versus misses (*M*) again for each stimulus type (*F*3_objects_ > *M*_objects_; *F*3_scenes_ > *M*_scenes_; *F*3_faces_ > *M*_faces_; *R*_objects_ > *M*_objects_; *R*_scenes_ > *M*_scenes_) and collapsed across type (*F*3 > *M* and *R* > *M*). For the recollection analyses, data from 16 participants were used as one participant did not give enough recollection responses at retrieval. The individual parametric and categorical contrasts of interest were entered into a one‐sample *t*‐test in the second‐level analysis treating participants as a random effect. All the produced SPM(t) maps, for all the analyses reported here, were initially thresholded at an uncorrected voxel level of *P* < 0.001 and clusters are reported as significant when at least 8 contiguous voxels were active unless noted differently. In the case of activations surviving a cluster‐wise FWE‐correction for multiple comparisons these are denoted separately.

### Multivariate Analyses

The multivariate analysis within the whole brain and the a priori ROIs were conducted using *Pattern Recognition for Neuroimaging Toolbox* (PRONTO, http://www.mlnl.cs.ucl.ac.uk/pronto/; Schrouff et al., 2013). In this multi‐voxel pattern analysis (MVPA) method the aim is to classify different experimental conditions within specified regions of interest, based on distributed patterns of activity across voxels. The a priori anatomical ROIs included the hippocampus, the perirhinal cortex (PRC), the entorhinal cortex (ERC), the parahippocampal cortex (PHC) and the amygdala. The PickAtlas Toolbox was used for the definition of each of these ROIs (Maldjian et al., 2003, 2004), with the exception of the PRC and ERC, which were identified individually for each participant using the probabilistic map created by Devlin and Price (2007) in combination with previously published anatomical criteria (Insausti et al., 1998). Each classification, as described below, was run separately for left and right structures as well as for the bilateral mask of each structure.

To investigate the two main questions of the present study, two sets of classification analyses were performed using in each case the condition‐specific fMRI beta images generated for each subject in the GLM analysis. First, to explore whether familiarity‐related brain responses within the different ROIs vary as a function of stimulus type, we used a Multiclass Gaussian Process Classification (GPC) (Rasmussen and Williams, 2006). This algorithm was applied to the *F*3 (strongly familiar) responses across the three types of stimuli (scenes vs. objects vs. faces). The same analysis was also conducted for the collapsed familiarity responses and is presented in the supplement (Supporting Information Fig. 1). To control for any potential systematic bias favoring the classification of one stimulus type over the other two types of stimuli in the multiclass GPC classification model, separate binary SVM (Support Vector Machine) classifications were also performed between all the possible stimulus combinations (i.e., scenes vs. objects; scenes versus faces; objects versus faces) and an aggregate classification accuracy (i.e., mean accuracy across all combinations) for each stimulus type was calculated. As the results produced from this approach are very similar to those from the main Multiclass GPC, they are not reported separately in the Results but can be found in Supporting Information Table 2. Finally, a separate analysis examined pattern classification for familiar stimuli using binary SVM between *F*3 responses and misses (*M*) separately for each stimulus type. This analysis, which is presented in Supporting Information Table 5, complemented the main GCP classification reported in the Results below and produced similar findings to it. However, any discrepancies are noted and discussed (see Results and Discussion).

To approach the second aim of this study regarding the role of the hippocampus in coding for recollection and/or equally strong familiarity, a second set of classification analyses compared classification performance for *F*3 and *R* responses versus misses (*F*3 vs. *M* and *R* vs. *M*) collapsed for the three types of stimuli using a binary SVM (Support Vector Machine) algorithm. Finally, to explore any differences in the classification outcome for *R*_scenes_ and *R*_objects_ in the hippocampus, a separate binary SVM model was also run. Each classification was performed separately for each ROI and included all voxels within each ROI (no feature selection was used). In these classification analyses the data were mean centered and a leave‐one‐subject‐out (LOSO) cross‐validation procedure was performed to ensure independence between training and test data sets. Specifically, this cross‐validation procedure involves repeated repartitioning of the training and test data to derive an estimate of the accuracy and the generalization error of the model across subjects. This group analysis, treating subjects as a random factor, enables the generalization of the classification results to the population. Statistical significance of the classifications within each ROI was tested using permutation testing (1000 permutations) for each model (classifier).

## RESULTS

### Behavioral Results

Memory accuracy [Hit rate/(Hit rate + FA rate)] for familiarity and recollection responses is presented in Figure 1B. The 3 × 3 ANOVA with stimulus type (objects, faces, scenes) and familiarity strength (*F*1, *F*2, *F*3) as the within‐subjects factors showed significant main effects of type (*F*_2,30_ = 3.44, *P* = 0.045) and strength (*F*_2,30_ = 150.99, *P* < 0.001). Pairwise comparisons revealed higher familiarity accuracy for objects than faces (*P* = 0.007), but no other difference across the three types, and increased memory accuracy with increased familiarity strength (*F*3 > *F*2 > *F*1; all *P*s < 0.001). Importantly, as presented in Figure 1B, *F*3 responses were characterized by matched memory accuracy with *R* responses, across all stimuli types collapsed (M_F3_ = 0.90, SD_F3_ = 0.09 and *M*_R_ = 0.90, SD_R_ = 0.13; *t* < 1) and separately for scenes (M_F3_ = 0.91, SD_F3_ = 0.08 and *M*_R_ = 0.90, SD_R_ = 0.26; *t* < 1) and objects (M_F3_ = 0.93, SD_F3_ = 0.10 and *M*_R_ = 0.90, SD_R_ = 0.18; *t* < 1). Recollection responses for faces were very rare and therefore are not evaluated separately.

The 3 × 3 ANOVA on the RTs showed a significant main effect of stimulus type (*F*_2,30_ = 54.31, *P* < 0.001), with longer latencies for scenes (1943 ms; SD = 42.61 ms) than both objects (1620 ms; SD = 39.52 ms) and faces (1656 ms; SD = 56.43 ms; both *P* < 0.001) and a significant effect of strength (*F*_2,30_ = 8.56, *P* = 0.001), indicating shorter latencies for more confident responses (*F*1 > *F*2 > *F*3). Matched RTs characterized recollection and *F*3 responses across all stimulus types collapsed (*F*3 = 1577 ms, SD = 213; *R* = 1653 ms, SD = 284 ms) and separately for scenes (*F*3_scenes_ = 1807 ms, SD = 281 ms; *R*_scenes_ = 1932 ms, SD = 421 ms) and objects (*F*3_objects_ = 1467 ms, SD = 230 ms; *R*_objects_ = 1537 ms, SD = 240 ms; all *t* < 1).

### fMRI Results

#### Material‐specific familiarity effects within the medial temporal lobes

In the univariate analysis, parametric increases or decreases across familiarity strength (from F0 to *F*3) were analyzed separately for scene, face and object stimuli (see Methods). Shared activation patterns within the MTL across all stimulus types as well as material‐specific familiarity activations for each type of stimulus were explored. In the conjunction analysis, no overlapping MTL region was found to respond to familiarity for the three types of stimuli. Instead, only material‐specific responses in the MTL were found during familiarity decisions. This means that structures of the MTL only provide material‐specific support when involved in familiarity decisions.

**Figure 2:**
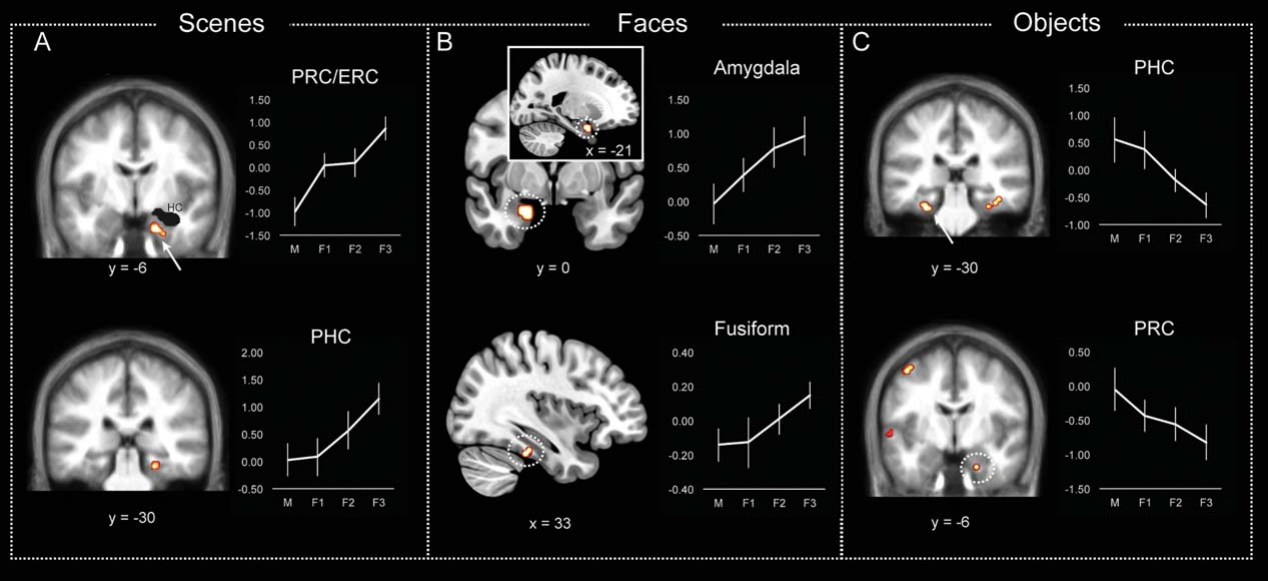
Material‐specific familiarity effects for (A) scenes, (B) faces and (C) objects in the MTL, the amygdala and the fusiform gyrus. Error bars show the standard error of the mean. [Color figure can be viewed at wileyonlinelibrary.com]

Specifically, a variety of familiarity responses across the three stimulus types emerged within the MTL. As shown in Figure 2, the right PHC (BA 35; *x* = 24, *y* = −30, *z* = −12 and *x* = 21, *y* = −33, *z* = −12, 21 voxels; Fig. 2A) and a cluster within the bilateral ERC and PRC (BA 28/35; only the right cluster survived the exclusive mask; *x* = 21, *y* = −6, *z* = −30, 9 voxels) increased their activity across familiarity strength selectively for the scene stimuli. In contrast, unique parametric deactivation patterns for object stimuli (Fig. 2C) were found within the left PHC (BA 36; *x* = −27, *y* = −30, *z* = −18, 5 voxels) as well as in an area of the anterior PRC/ERC (*x* = 21 *y* = −6 *z* = −27) at a lower threshold (*P* < 0.001, uncorrected; 2 voxels). Finally, a cluster within the left amygdala (including the dorsal ERC; BA 34; *x* = −21, *y* = 0, *z* = −24; 35 voxels) and another cluster within the fusiform gyrus (BA 20; *x* = 33, *y* = −36, *z* = −24; 17 voxels) uniquely responded to face familiarity (Fig. 2B). Importantly, no shared or selective familiarity response was found in the hippocampus for any of the three types of stimuli even at a lower threshold (*P* < 0.01, uncorrected).

#### MVPA analysis

**Figure 3:**
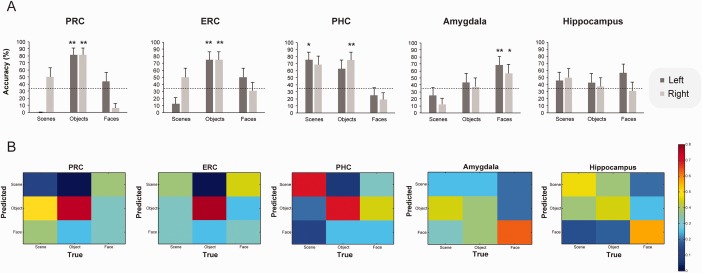
(A) Multivariate pattern recognition classification accuracy and (B) confusion matrices for the classification of strong familiarity (*F*3) responses within the perirhinal cortex (PRC), the entorhinal cortex (ERC), the parahippocampal cortex (PHC), the amygdala and the hippocampus for each stimulus type. Dashed lines in the graphs mark chance classification. Error bars indicate the standard error of the mean across participants. * *P* < 0.05; ** *P* < 0.01. *P*‐values were obtained through permutation testing with 1000 permutations.

Multivariate classification was conducted for the whole brain and a priori ROIs including the hippocampus, the PRC, the ERC, the PHC, and the amygdala. A multi‐class Gaussian Process Classification (GPC) algorithm was trained to classify *F*3 (strongly familiar) responses for scenes, objects and faces across participants (see Methods; see also Supporting Information Table 2 for an alternative analysis). The classification profile, in terms of classification accuracy and classification errors, for each anatomical ROI is summarized in Figure 3 and in Supporting Information Table 3. The whole brain analysis yielded a significant accuracy of 60.4% (*P* = 0.01) and significant classification accuracy for scenes (accuracy = 56.3%, *P* = 0.05), objects (accuracy = 68.8%, *P* = 0.05) and faces (accuracy = 56.3%, *P* = 0.03). This indicates that activation patterns within the whole brain successfully discriminated *F*3 responses across the three types of stimuli. In the hippocampus, classification accuracy for *F*3 responses to scenes, objects and faces was low and not significant (Fig. 3), whereas in the PRC there was a selective significant classification for objects versus faces and scenes (whole PRC: accuracy = 75%, *P* = 0.01; Left PRC = 81.3%, *P* = 0.01 and Right PRC = 81.3%, *P* = 0.01; Fig. 3). Similar to the PRC, familiar objects, versus the other two stimulus types, were significantly classified in the ERC (whole ERC: accuracy = 75%, *P* = 0.02; Left PRC = 75%, *P* = 0.01 and Right PRC = 75%%, *P* = 0.01; Fig. 3). In the PHC, as shown in Fig. 3, significant classification accuracy was found for scenes in the whole PHC (accuracy = 68.8%, *P* = 0.02) and the left PHC (accuracy 75%, *P* = 0.02) and for objects in the right PHC (accuracy = 75%, *P* = 0.01). Similar results were obtained when partitioning the parahippocampal gyrus (including both PRC and PHC) into anterior, middle and posterior portions. The anterior aspect (corresponding to the PRC/ERC) showed selective classification sensitivity to object familiarity (accuracy = 68.8, *P* = 0.02). The posterior aspect (corresponding to the PHC) showed significant classification accuracy for both objects (accuracy = 81.3, *P* = 0.01) and scenes (accuracy = 68.8, *P* = 0.02), while the middle aspect of the gyrus, corresponding to the transitional zone between PRC and PHC, was also found to code for both object (accuracy = 68.8, *P* = 0.04) and scene familiarity (accuracy = 68.8, *P* = 0.04). In the amygdala (Fig. 3), classification accuracy was significant for faces within the left (accuracy = 56.3, *P* = 0.05) and the right amygdala (accuracy = 62.5%, *P* = 0.04) but not for the other two stimulus types. Similar multivariate classification findings were obtained when the same analysis was conducted for the familiarity responses collapsed across the three levels of strength (Supporting Information Fig. 1) and when *F*3 responses were classified relative to misses separately for scenes, objects and faces (Supporting Information Table 5). The only difference between the main analysis, as reported above, and the one classifying familiar (*F*3) versus missed stimuli (reported in Supporting Information Table 5), was the accurate classification of familiar faces in the left ERC (accuracy = 68.75, *P* = 0.02).

#### Selective hippocampal response to recollection

**Figure 4:**
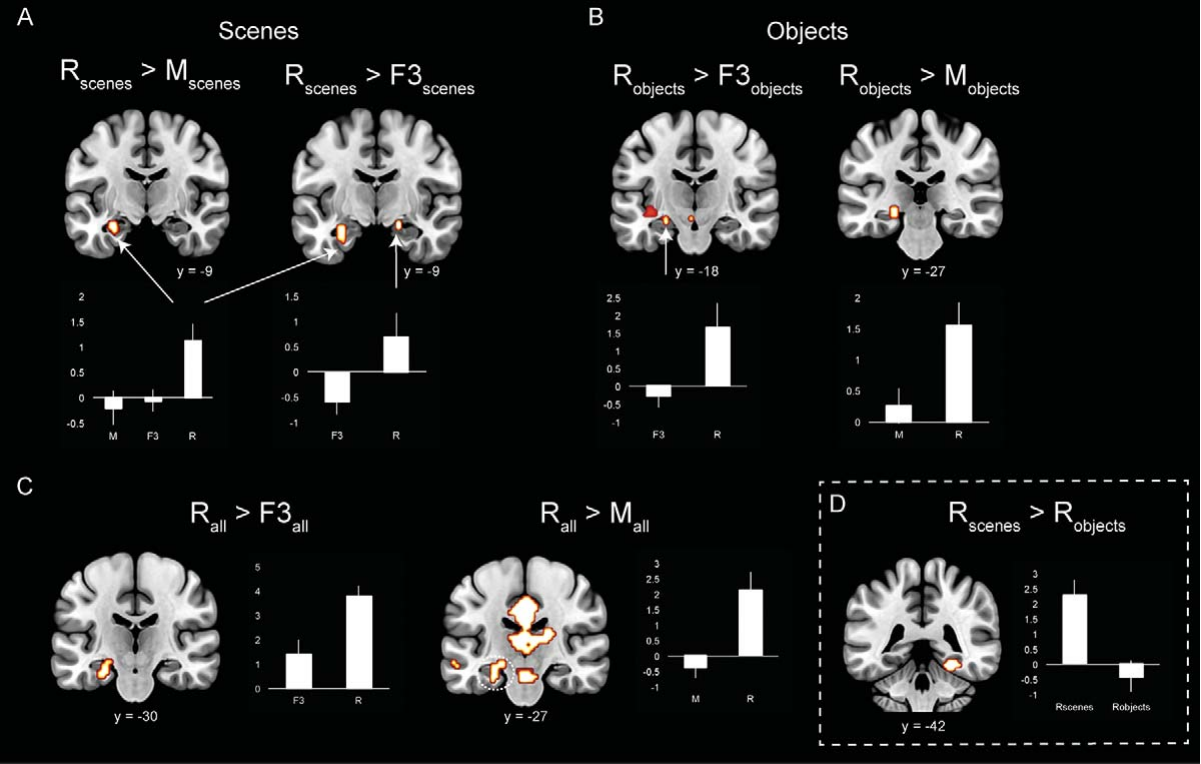
Recollection selective response in the hippocampus for (A) scenes, (B) objects and (C) collapsed across scenes, objects and faces. (D) Selective parahippocampal cortex effect for *R*_scenes_ versus *R*_objects_. Error bars show the standard error of the mean. [Color figure can be viewed at wileyonlinelibrary.com]

In the univariate analyses, as summarized in Figure 4, selective responses to *R* were found in the hippocampus in a series of contrasts between *R* versus accuracy‐matched *F*3 responses and *R* versus misses (*M*). Specifically, the bilateral hippocampus (Left: *x* = −15, *y* = −30, *z* = −6, *P* < 0.001, 21 voxels; Right: *x* = 18, *y* = −15, *z* = −15, *P* < 0.001, 9 voxels) selectively responded to *R* versus *F*3 responses collapsed across the three stimulus types (Fig. 4C). Hippocampal activation was also found when *R* > *F*3 contrast was conducted separately for objects (*R*_objects_ > *F*3_objects_: *x* = −21, *y* = −27, *z* = −6 and *x* = −24, *y* = −18, *z* = −15, *P* < 0.05 FWE‐corrected, 18 voxels; Fig. 4B) and scenes (*R*_scenes_ > *F*3_scenes_: *x* = −27, *y* = −9, *z* = −21, *P* < 0.001, 14 voxels and *x* = 18, *y* = −9, *z* = −15, *P* < 0.001, 10 voxels; Fig. 4A) denoting that the hippocampus has a material‐independent role in recollection. A direct contrast between *R*_objects_ and *R*_scenes_ (both *R*_objects_ > *R*_scenes_ and *R*_scenes_ > *R*_objects_) yielded only a significant cluster in the PHC (*x* = 27, *y* = −42, *z* = −15, *P* < 0.001, 25 voxels) responding more to *R*_scenes_ than *R*_objects_ (Fig. 4D), while no differential activation for either *R*_objects_ or *R*_scenes_ was found in the hippocampus.

To further validate the selectiveness of the hippocampal response to *R* relative to strength‐matched familiarity (*F*3), separate analyses were run contrasting *R* versus misses (*M*) and *F*3 versus *M*, both collapsed across stimulus type and separately for objects and scenes (and faces for *F*3_faces_ versus *M*_faces_). As shown in Figure 4, recollection responses to scenes, objects and collapsed across scenes, objects and faces, resulted in greater activations within the hippocampus relative to misses (*R*_scenes_ > *M*_scenes_: *x* = −27, *y* = −9, *z* = −21, *P* < 0.05, FWE‐corrected, 20 voxels; *R*_objects_ > *M*_objects_: *x* = −15, *y* = −36, *z* = 0 and *x* = −24, *y* = −27, *z* = −6, *P* < 0.001, 18 voxels; *R*_all_ > *M*_all_: *x* = −18, *y* = −27, *z* = −9 and *x* = −24, *y* = −6, *z* = −21, *P* < 0.05 FWE‐corrected, 60 voxels). In contrast, none of the contrasts between *F*3 and misses for each stimulus type, separately and collapsed, yielded any significant activation within the hippocampus even at a considerably lower threshold (*P* < 0.01, uncorrected).

Overall, these findings stress that the hippocampus selectively supports recollection (versus familiarity) and it does so in a non‐material specific fashion. To further explore the degree of overlap of the recollection activations in the hippocampus for the two types of stimuli (i.e., objects and scenes), two conjunction analyses were further conducted. The first one was performed between *R*_objects_ > *F*3_objects_ and *R*_scenes_ > *F*3_scenes_, while the second one between *R*_objects_ > *M*_objects_ and *R*_scenes_ > *M*_scenes_. Both conjunction analyses (see Supporting Information Fig. 2) confirmed that overlapping areas within the hippocampus respond to recollection for objects and scenes (*R* > *F*3 conjunction: left hippocampus *x* = −24, *y* = −18, *z* = −21, *P* < 0.05, FWE‐corrected, 20 voxels and right hippocampus *x* = 24, *y* = −21, *z* = −15, *P* < 0.001, 8 voxels; *R* > *M* conjunction: right hippocampus *x* = 24, *y* = −27, *z* = −12, *P* < 0.05, FWE‐corrected, 13 voxels).

#### MVPA analysis

**Figure 5:**
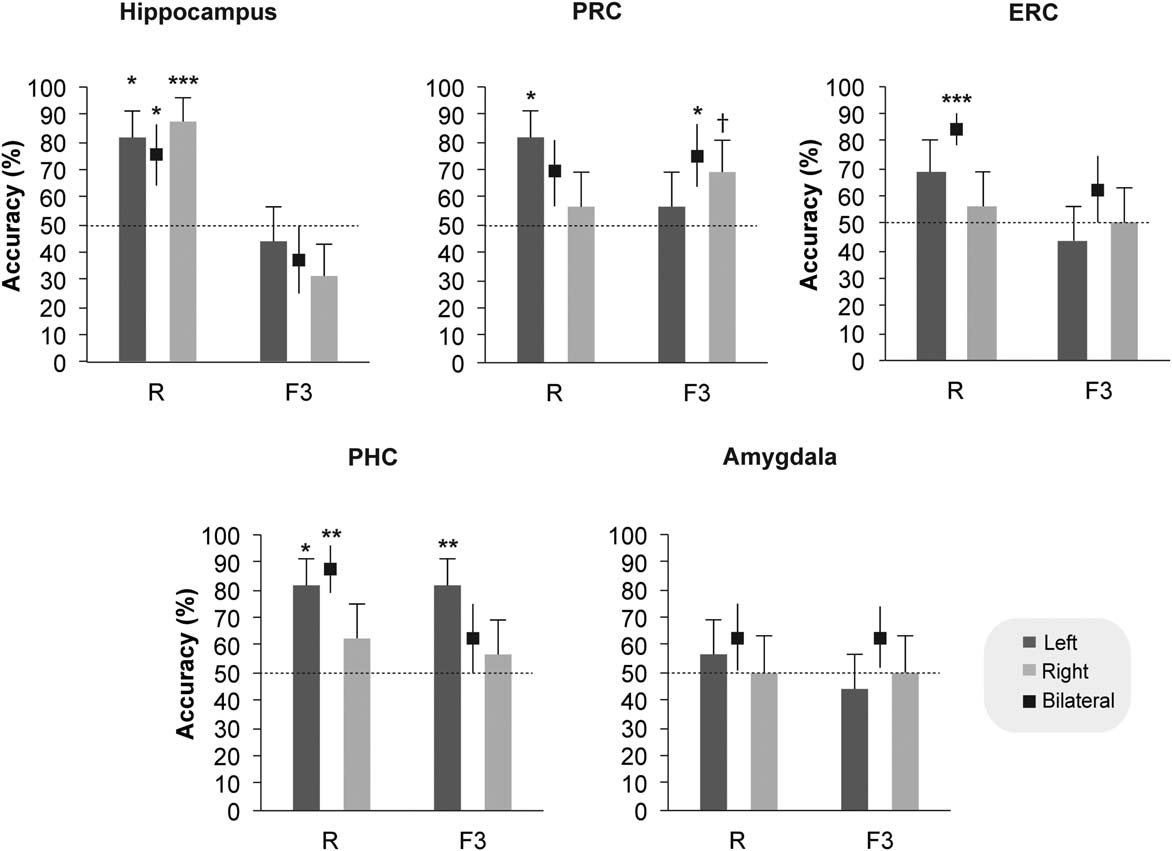
Multivariate pattern recognition classification accuracy for *R* and *F*3 responses in the hippocampus, the perirhinal cortex (PRC), the entorhinal cortex, the parahippocampal cortex (PHC) and the amygdala. Error bars indicate the standard error of the mean across participants. * *P* < 0.05; ** *P* < 0.01; *** *P* < 0.001; † *P* = 0.08 (trend). *P*‐values were obtained through permutation testing with 1000 permutations.

To compare classification performance for *F*3 and *R* responses within each ROI and specifically within the hippocampus, binary SVM analyses were used (see Methods) to classify *F*3 and *R* responses versus misses collapsed across the three types of stimuli (Fig. 5 and Supporting Information Table 4). As shown in Figure 5, classification accuracy for *R* responses was significantly above chance within both the left (accuracy = 81.3%, *P* = 0.02) and the right hippocampus (accuracy = 87.5%, *P* = 0.001). In contrast, *F*3 responses were classified very poorly in the hippocampus producing low and non‐significant classification accuracy (Fig. 5). Classification within the PRC and the PHC was significant for both *F*3 and *R* responses. Specifically, classification accuracy was significant in the whole PRC for *F*3 responses (accuracy = 75%, *P* = 0.03), whereas *R* responses were reliably classified only in the left PRC (accuracy = 81.3%, *P* = 0.02). In the PHC, classification within the left PHC yielded significant classification accuracy for both *F*3 (accuracy = 81.3%, *P* = 0.007) and *R* (accuracy = 81.3%, *P* = 0.012), while within the whole PHC significant classification accuracy was found only for *R* responses (accuracy = 87.5%, *P* = 0.003). In the ERC, classification within the whole ERC mask was significant for *R* (accuracy = 84.4, *P* = 0.001), but no other significant classification outcome for *R* or *F*3 was found either in the left or the right ERC. Finally, classification accuracy in the amygdala was low and non‐significant for both *F*3 and *R* responses.

An SVM analysis was also performed to compare *R*_objects_ and *R*_scenes_ specifically in the hippocampus. Consistent with the conjunction analyses reported above for scene and object recollection and the lack of any difference in the hippocampus when contrasting *R*_scenes_ and *R*_objects_, this analysis yielded a very low overall model accuracy in the hippocampus (accuracy = 56.7%, *P* = 0.26) denoting the very poor discrimination between recollection for objects and scenes within the hippocampus. This is consistent with the hippocampus supporting recollection in a non‐material specific way. Indeed, when *R*_objects_ and *R*_scenes_ were examined separately, relative to misses, they produced equally good classification outcomes in the hippocampus (*R*_objects_: accuracy = 62.5%, *P* = 0.05; *R*_scenes_: accuracy = 73.3%, *P* = 0.02).

## DISCUSSION

In this fMRI study we employed a recognition memory paradigm to explore the interaction between stimulus content and the kind of memory engaged at retrieval. Our primary aim was to investigate whether familiarity for different kinds of visual stimulus is mediated by a common MTL neural network or whether there are at least partially non‐overlapping MTL networks mediating familiarity for different kinds of stimulus. This question is particularly important because different models of MTL functional organization currently make different predictions about the contribution of MTL structures in familiarity‐based recognition for different stimulus types (see Introduction). The findings presented here point toward a degree of specialization, with respect to stimulus type, within the MTL structures (including the neocortical structures and the amygdala) for familiarity. On the other hand, overlapping regions of the hippocampus mediated recollection, regardless of the kind of visual stimulus to which it related. However, no response to familiarity was found in the hippocampus, even when recollection was contrasted to equally accurate familiarity. These findings have important implications for current theories of recognition memory and for evaluating the extent of functional specialization within the MTL. These are further discussed below.

### Stimulus Content and Familiarity‐Based Recognition: Selectivity Within the MTL

As discussed in the Introduction, different models of recognition memory make different predictions with respect to the role of PHC in supporting familiarity memory with some models stressing its role in supporting recollection (Diana et al., 2007) and others stressing its role in familiarity‐based recognition (Montaldi and Mayes, 2010). The MTL cortices and specifically PRC, ERC and PHC were found in the present study to respond to stimulus familiarity for object and scene stimuli but not for faces. The parametric analyses identified regions that increased or decreased their activity as a function of the reported familiarity strength. The identified regions are assumed to have a special role in familiarity‐based recognition, as their activity systematically correlates with the level of reported familiarity (Montaldi et al., 2006; Kafkas and Montaldi, 2014). The PHC showed significant increases in activity with increased familiarity strength for scenes, but decreased activity with increased familiarity strength for objects. The same pattern was observed in the PRC/ERC with increases in activity tracking reported familiarity strength for scenes, but decreases in activity accompanying increased familiarity strength for objects. These findings indicate that both PHC and PRC/ERC have a role in familiarity‐based recognition at least for objects and scenes, but not for faces. Nevertheless, the differential direction of the activation modulation with familiarity strength, within the PHC and PRC/ERC depending on the type of stimulus—activation pattern for scenes and deactivating patterns for objects—may suggest that familiarity‐based recognition for both objects and scenes triggers different computations within the MTL cortices. Indeed, to the extent that feelings of familiarity can be driven by a number of underlying processes (for a discussion of this see Montaldi and Mayes, 2010; Kafkas and Montaldi, 2014), then the computations performed by the MTL cortices could differ; for example, relating to repetition suppression, familiarity strength or evaluation of this strength.

The multivoxel classification analyses, a more sensitive way of capturing activity changes across multiple voxels within a region (Norman et al., 2006), indicated a further degree of specialization, at least in the PRC and ERC. Specifically, the PRC and ERC coded familiarity‐based recognition selectively for objects, whereas the PHC coded familiarity signals for both objects and scenes. Partitioning the MTL cortex (i.e., dividing parahippocampal gyrus) into anterior, middle and posterior aspects revealed consistent results, with the anterior aspect (corresponding to the ERC and PRC) showing selective classification sensitivity to object familiarity and the posterior aspect (corresponding to the PHC) showing accurate classification for both objects and scenes. The middle aspect of the gyrus, corresponding to the transitional zone between PRC and PHC, was also found to code for both object and scene familiarity. This finding partially agrees with previous evidence showing a gradient in functional specialization within the MTL cortex with respect to object and scene information, which does not follow strict boundaries between PHC and PRC (Litman et al., 2009; Staresina et al., 2011). In these studies, as in our findings, the most anterior aspect of the MTL cortex responded to object stimuli, whereas the middle transitional zone of the cortex responded to both object and scene information. This transitional zone between PRC and PHC has also recently been found to have unique functional and anatomical characteristics in relation to the more anterior or posterior portions of the parahippocampal gyrus (Zhuo et al., 2016). Nevertheless, contrary to the selective response to scene stimuli in the posterior MTL cortex in previous studies (Litman et al., 2009; Staresina et al., 2011), our findings show that even in the posterior aspect of the gyrus (and indeed even in the posterior PHC), the activation patterns were sensitive to both object and scene familiarity.

Face familiarity, on the other hand, did not lead to univariate parametric activity, and did not result in an accurate classification outcome, in the MTL cortices or the hippocampus. However, the amygdala was found in both analyses (univariate and multivariate classification) to selectively code for face familiarity. The amygdala cluster in the univariate analysis also included voxels (9 out of 35 active voxels) falling within the ERC (BA 34). This finding may agree with recent evidence that areas within the anterior temporal lobe (including portions of the ERC and PRC) have a role in face discrimination and identification (O'Neil et al., 2009; Nestor et al., 2011; Nasr and Tootell, 2012; Rossion et al., 2012; Von Der Heide et al., 2013; but see Axelrod and Yovel, 2015). However, when examined separately, the multivariate analysis within the ERC favored the classification of object, but not faces, similar to the pattern observed in the PRC, whereas the amygdala ROI clearly favored the classification of familiar face stimuli. It is possible, therefore, considering the proximity of the two structures, that the observed univariate parametric activation in the ERC is driven predominantly by the amygdala activation. However, in one alternative MVPA analysis (Supporting Information Table 5), as note in the Results, when classifying *F*3 relative to misses for each stimulus type left ERC was found to accurately and significantly discriminate familiar faces. Therefore, these findings cannot exclude the possibility that a portion of the ERC – especially the dorsal BA 34 area – has a role in face familiarity along with the amygdala.

It has been reported before that the amygdala interacts with the hippocampus to promote encoding of emotional (Dolcos et al., 2005; LaBar and Cabeza, 2006), as well as non‐emotional information (Babiloni et al., 2009), and that successful memory encoding of item information activates the amygdala (Kensinger and Schacter, 2006). Selective amygdala activation to nominally neutral faces has also been reported in novelty detection tasks (Schwartz et al., 2003; Wright et al., 2003) and when faces and scenes are contrasted (Balderston et al. 2011). Our findings further suggest that the amygdala has a selective role in supporting familiarity‐based recognition for faces, even when the faces were not overtly emotionally arousing. Therefore, contrary to the view that the neocortical MTL structures (and especially the PRC) supports familiarity‐based recognition for every stimulus type (Aggleton and Brown, 1999; Montaldi and Mayes, 2010), our findings show that familiarity decisions for faces is not accomplished in the MTL cortices but predominantly in the subcortical amygdala, although the dorsal ERC may also have a role.

One contentious issue with respect to this proposal, is that previous neuropsychological evidence (Taylor et al., 2007; Mundy et al., 2013) suggests that face learning and recognition should rely on the integrity of the MTL cortices. However, in these studies, lesions within the MTL were not confined to the parahippocampal gyrus but normally extended into the amygdala. Furthermore, sparing of the MTL neocortical regions and the amygdala when the hippocampus was damaged, resulted in normal recognition performance when faces were involved (Mayes et al., 2002; Bird et al., 2007). Consistent with this suggestion, lesions of the amygdala in rats have been reported to selectively result in impaired familiarity for odors (Farovik et al., 2011). Odors can be seen as carrying important social cues for rats in the same way as faces signify an important social cue to humans. Indeed, in humans, selective amygdala damage has not only been associated with deficits in facial expression recognition (especially fear; Adolphs et al., 1999; Mattavelli et al., 2014), but also with impaired learning of new faces (Young et al., 1995). Therefore, a critical role of the amygdala in familiarity‐based recognition for faces is convergently supported by both lesion and fMRI evidence.

The lack of PRC sensitivity to face familiarity (in both univariate and multivariate analyses) may indicate that the role of PRC in face processing is limited to face discrimination and identification (as noted in previous studies; e.g., Rossion et al., 2012; Olson et al., 2015) without, nevertheless, contributing to familiarity assessment. However, although the present study was not designed to explore face perception or discrimination, even when all the face stimuli were contrasted with either objects or scenes, irrespective of their memory status, no noticeable activation in PRC for faces even at a very low threshold (*P* < 0.01, uncorrected) was found. Instead, other, more posterior areas of the inferior temporal lobe (including the fusiform gyrus), were activated by faces (for a similar lack of activations in the anterior temporal areas for faces see Axelrod and Yovel, 2015). Therefore, these findings are not consistent with a role of the PRC in face discrimination.

It is possible, however, that any potential role of the PRC, as well as the rest of the anterior temporal lobe, in face discrimination, as has been proposed in a few recent studies (O'Neil et al., 2009; Nestor et al., 2011; Nasr and Tootell, 2012; Rossion et al., 2012; Von Der Heide et al., 2013), is contingent on the nature of the perceptual discrimination tasks that have been used in previous studies as opposed to a memory task as employed here. However, O'Neil et al., (2009) and Martin et al. (2013; see also Martin et al., 2016) also using memory tasks (memory discrimination and familiarity‐based recognition tasks respectively), reported PRC activations for faces. As noted above, although no face familiarity signal was identified in the PRC in the present study, another anterior temporal area (the left ERC) was found to have a potential role in face familiarity, as indicated in the univariate activation patterns (clustered with the amygdala activation) and in one of the additional MVPA analyses reported in Supporting Information Table 5. Could this discrepancy between previous studies that have identified face familiarity signal in the PRC (O'Neil et al., 2009; Martin et al., 2013) and the present findings be explained by differences in the anatomical definition of PRC and ERC ROIs? We do not consider this to be a valid explanation as an ROI‐led analysis in the present study was used in the MVPA analysis and not in the univariate analysis, where the ERC activation to face familiarity was identified too (within the amygdala cluster). Also, although the ROI definition in the present study was based on probabilistic maps, the separation of the ERC and PRC was performed at the individual levels following established landmark‐based criteria (see Methods). Therefore, our findings show that areas within the anterior temporal lobe (ERC and amygdala) carry face familiarity information but such signal was not identified in the PRC. Further studies, therefore are needed to evaluate the contribution of different tasks in modulating the activity within areas along the tip of the collateral sulcus in face familiarity.

Our findings, only partially support the previously proposed functional division between PRC and PHC as specializing in processing object and scene information, respectively (Lee et al., 2005; Davachi, 2006; Eichenbaum et al., 2007; Montaldi and Mayes, 2010; Staresina et al., 2011; Watson et al., 2012). Rather the PHC appears to have a more general role in coding familiarity, at least for objects and scenes. In a previous MVPA study, Diana et al. (2008) reported non‐stimulus selective classification of pictorial stimuli (scenes, objects, faces and toys) in the PHC and Liang et al. (2012), reported significant classification of various stimulus types (faces, scenes and words) within both PHC and PRC. Furthermore, Bar et al. (2008; see also Aminoff et al. 2007, 2013) have proposed that the role of the PHC is not limited to processing scene or place information but in coding contextual associations for spatial and non‐spatial stimuli (for a similar discussion see also Montaldi and Mayes, 2010). It is, therefore, reasonable to propose that functional divisions within the MTL strictly limited to a division between scenes and objects cannot explain the breadth of findings.

One unique aspect of the present study is the emphasis on investigating the role of the PRC, ERC and PHC selectively in familiarity‐based recognition and not on visual discrimination or recollection memory retrieval as in all previous studies that have explored material‐specificity effects in the MTL (Awipi and Davachi, 2008; Diana et al., 2008; Litman et al., 2009; Preston et al., 2010; Duarte et al., 2011; Staresina et al., 2011, 2013; Liang et al., 2012). However, as perception and recognition memory coincide and, in many cases, it is difficult to experimentally separate them, one important question in relation to our findings is to what extent the material specificity found for familiar stimuli in the MTL is driven by perception more than memory for the items. This is more relevant in the case of the MVPA approach as, in this case, a classification analysis across the three types of familiar stimuli may be attributed to perceptual factors and not necessarily to the sensitivity of these structures to the type of memory (i.e., familiarity). There are, however, two important points that speak against this interpretation. First of all, there is a striking agreement between the univariate and the multivariate effects in the MTL. Taking this into account, a perceptual explanation cannot account for the fact that the areas that are reported as selectively coding for familiarity for the different types of stimuli, also responded in a parametric way tracking increases in reported familiarity strength. In this case, an area that increases its activity from weak to strong familiarity for a type of stimulus (e.g., objects) does so because it detects changes in the reported familiarity and not because it responds to the perceptual characteristics of objects. Secondly, when running the classification analysis to compare strong familiarity (*F*3) to misses (*M*) for each type of stimulus separately (Supporting Information Table 5), similar findings to those reported in the main analysis are revealed. In this case the type of stimulus is the same between *F*3 and *M* and therefore the successful classification and discrimination of these two responses (*F*3 vs. *M*) cannot be attributed to the stimulus type, but only to the existence or not of familiarity for the stimulus

The findings presented here provide direct evidence that the PHC supports familiarity‐based recognition at least for objects and scenes, along with a more specialized role of the PRC and ERC in object familiarity. These findings challenge the traditional view of a specialized role of PHC in supporting memory for spatiotemporal types of contexts, such as scene stimuli. That said, one possibility, which is worth considering, is that the MTL cortices may engage in familiarity‐based recognition for different types of object stimuli or different object properties (such as size, contextual reference etc.). Indeed, some evidence for this comes from a recent study (Martin et al., 2013) in which familiarity responses in the PHC were selectively identified for buildings, but also from studies linking PHC to specific properties of scene stimuli which may also contain objects (Bar et al., 2008). This issue merits further investigation in order to understand which specific object dimensions/properties during familiarity decisions may be selectively processed in the PHC.

Related to this point, in one of our previous studies (Montaldi et al., 2006) in which familiarity memory for scenes was explored, PRC (but not PHC) was found to respond to scene familiarity. The difference in relation to the role of PHC in scene familiarity between the present findings and the ones reported in Montaldi et al., (2006), may be attributed to the characteristics of the scene stimuli used in the two studies. In the present study landscape images with very few central objects were used promoting, therefore, processing of broader visuospatial contexts. In contrast, Montaldi et al., (2006) used scene stimuli that constituted unique events incorporating object information too. Therefore, the role of the PRC in the earlier study may have been driven predominantly by object information within the scenes or by processing scenes more as objects and not as complex visuospatial arrays. Indeed, the direction of the familiarity effects in the prior study (Montaldi et al., 2006) is the same as the direction of the deactivation patterns in the PRC for object stimuli as found in the present experiment.

The findings also provide clear evidence for the role of ERC in familiarity‐based recognition, at least for object stimuli and possibly for faces. The role of this structure appears to be very similar to that of the PRC in familiarity discrimination as evident from the very similar classification outcomes within the two structures (see Fig. 3). Although, most models of recognition memory do not make any explicit predictions about the role of ERC in familiarity‐based recognition, our findings, along with other fMRI and lesion evidence (Ranganath et al., 2004; Yonelinas et al., 2007; de Vanssay‐Maigne et al., 2011; Brandt et al., 2016), indicate that its role in mediating familiarity memory for at least some kinds of stimulus information may be more important than previously appreciated.

One final note is that the degree of functional specialization within the MTL cortices and the amygdala in supporting familiarity memory, as found here, should be consistent with the cytoarchitectural profiles of these regions and the inputs they receive. Indeed, as described in the Introduction, the MTL cortices are characterized by similar cytoarchitecture and therefore, it is plausible that they perform similar computations with respect to stimulus familiarity. However, they also receive different inputs from other cortical areas, which may constrain the type of stimuli they process, consistent with the findings from the present study with the more selective role of the PRC and ERC in processing familiarity for object stimuli. On the other hand, the subcortical amygdala has a unique and complex cytoarchitecture (Pitkänen, 2000; Pitkänen and Kemppainen, 2002), radically different from the adjacent MTL cortices. It is, therefore, plausible that the type of familiarity processing that the amygdala applies to the stimuli it processes (e.g., faces), is also radically different from the kind of familiarity computed in the neocortical MTL regions. Such a discrimination implies that familiarity may be a multi‐process kind of memory, with differences expressed within the MTL or even in extra‐MTL regions, a suggestion that merits further investigation.

### The Hippocampus Supports Recollection, but Not Familiarity, Irrespective of Stimulus Type

A secondary aim of the present study was to explore more closely whether the hippocampus has a selective role in recollection, when compared to equally accurate familiarity, and whether this selective role applies to all three different types of cuing items (objects, faces and scenes). As described in the Introduction, although strong neuroimaging evidence supports the selective role of the hippocampus in recollection, but not in familiarity (for reviews see Eichenbaum et al., 2007; Skinner and Fernandes, 2007; Montaldi and Mayes, 2010; Rugg and Vilberg, 2013), a counterproposal has challenged this evidence on the basis of a methodological confound (Squire et al., 2007; Wixted et al., 2010). According to this argument, when strong memories are produced (in terms of reported confidence and accuracy), irrespective of whether the basis is familiarity, recollection or a combination of the two, the hippocampus will be active.

The results presented here are inconsistent with this prediction. First, activity in the hippocampus was not modulated by familiarity strength for any of the three kinds of stimulus used in the present study. Second, the hippocampus responded to recollection for all stimulus types collapsed and separately for objects and scenes when compared to misses and equally accurate familiarity. This was also complemented by a reliable selective classification of recollection responses in the hippocampus, but not *F*3 (strongly familiar) responses. These patterns of results are not compatible with the predictions suggested by the strength confound view, but join the previous evidence for a selective role of the hippocampus in supporting recollection (Yonelinas et al., 2005; Montaldi et al., 2006; Cohn et al., 2009; Montaldi and Mayes, 2010; Kafkas and Montaldi, 2012; Rugg et al., 2012; for a recent review see Rugg and Vilberg 2013).

The findings further stress that this contribution of the hippocampus in supporting recollection is not material‐specific (for comparable results see Konkel and Cohen, 2009; Duarte et al., 2011; Staresina et al., 2011, 2013) but applies to at least three types of visual stimuli – scenes, faces and objects. With respect to face recollection, we were unable to explore the hippocampal response selectively for faces because of the small number of recollection responses to faces. This may stem from the high degree of similarity characterizing face stimuli, reaching the limits of pattern separation computation in the hippocampus (Kim and Yassa, 2013), coupled with the use of a shallow encoding task, which may have further hindered the formation of contextual associations at encoding. Nevertheless, hippocampal activity related to recollection relative to equally accurate familiarity was also found in the collapsed analyses across the three types of stimuli. However, this finding cannot exclude the possibility that the hippocampal activity is driven to a greater extent by recollection in the case of objects and scenes. Therefore, the proposed role of the hippocampus in supporting face recollection requires further investigation.

It is worth noting here that despite the aforementioned sensitivity of the PHC to familiarity‐based recognition for both scenes and objects, a recollection effect within the posterior PHC for scene stimuli was also isolated when contrasting scene recollection to object recollection (Fig. 4D). Considering the dense anatomical connectivity between the hippocampus and the posterior PHC (Suzuki and Amaral, 1994) and the selective recollection response within the hippocampus, one possible interpretation is that scene/context‐selective areas in the posterior PHC provide scene‐selective input to the recollection signals computed in the hippocampus. The proposal that the recollection effect in the posterior PHC is probably stimulus‐driven is further reinforced by the fact that this effect vanishes when contrasting scene recollection with scene familiarity. This means that the activation in the posterior PHC is not critical to recollection but stems from the presentation of familiar scene stimuli. Therefore, although this finding is compatible with the view that the PHC supports recollection of contextual details (Diana et al., 2007), this probably only occurs when familiar scene stimuli act as cues for recollection. Related to this finding, recollected stimuli were classified reliably in the MTL cortical areas (PRC, PHC and ERC) in the pattern classification analysis. As noted above for PHC, this finding may relate to the fact that most (possibly even all) recollected stimuli are potentially familiar too.

## CONCLUSIONS

Taken together, familiarity‐based recognition responses within the MTL were found to be material‐specific, with PRC and ERC responding to object familiarity and the PHC to both object and scene familiarity. The amygdala was found to have a selective role in familiarity‐based recognition for faces, whereas the adjacent hippocampus did not respond to stimulus familiarity for any of the three types of stimuli employed in the present study, in either the univariate and multivariate analysis. In contrast, the hippocampus was found to have a non‐material specific role in recollection, even when compared to strength‐matched familiarity. These findings illustrate the prominent role of the MTL neocortical areas and the amygdala in supporting familiarity‐based recognition and show that this role is constrained by the inputs these structures receive and the content of stimulus category they process. Overall, the findings point to a degree of specialization within the MTL that respects whether familiarity or recollection is active and (secondarily) the kind of stimulus that is recognized as familiar.

## Supporting information

Supporting Information

## References

[hipo22683-bib-0001] Adolphs R , Tranel D , Hamann S , Young A , Calder A , Phelps E , Anderson A , Lee G , Damasio A. 1999 Recognition of facial emotion in nine individuals with bilateral amygdala damage. Neuropsychologia 37:1111–1117. 10509833 10.1016/s0028-3932(99)00039-1

[hipo22683-bib-0002] Aggleton JP , Brown MW. 1999 Episodic memory, amnesia, and the hippocampal, anterior thalamic axis. Behav Brain Sci 22:425–444. 11301518

[hipo22683-bib-0003] Aminoff E , Gronau N , Bar M. 2007 The parahippocampal cortex mediates spatial and nonspatial associations. Cereb Cortex 17:1493–1503. 16990438 10.1093/cercor/bhl078

[hipo22683-bib-0004] Aminoff EM , Kveraga K , Bar M. 2013 The role of the parahippocampal cortex in cognition. Trends Cogn Sci 17:379–390. 23850264 10.1016/j.tics.2013.06.009 PMC3786097

[hipo22683-bib-0005] Ashburner J. 2007 A fast diffeomorphic image registration algorithm. Neuroimage 38:95–113. 17761438 10.1016/j.neuroimage.2007.07.007

[hipo22683-bib-0006] Awipi T , Davachi L. 2008 Content‐specific source encoding in the human medial temporal lobe. J Exp Psychol Learn Mem Cogn 34:769–779. 18605867 10.1037/0278-7393.34.4.769 PMC2938959

[hipo22683-bib-0007] Axelrod V , Yovel G. 2015 Successful decoding of famous faces in the fusiform face area. PLoS One 10:e0117126‐ 25714434 10.1371/journal.pone.0117126 PMC4340964

[hipo22683-bib-0008] Babiloni C , Vecchio F , Mirabella G , Buttiglione M , Sebastiano F , Picardi A , Di Gennaro G , Quarato PP , Grammaldo LG , Buffo P , Esposito V , Manfredi M , Cantore G , Eusebi F. 2009 Hippocampal, amygdala, and neocortical synchronization of theta rhythms is related to an immediate recall during rey auditory verbal learning test. Hum Brain Mapp 30:2077–2089. 18819109 10.1002/hbm.20648 PMC6871026

[hipo22683-bib-0009] Balderston NL , Schultz DH , Helmstetter FJ. 2011 The human amygdala plays a stimulus specific role in the detection of novelty. Neuroimage 55:1889–1898. 21256226 10.1016/j.neuroimage.2011.01.034 PMC3062695

[hipo22683-bib-0010] Bar M , Aminoff E. 2003 Cortical analysis of visual context. Neuron 38:347–358. 12718867 10.1016/s0896-6273(03)00167-3

[hipo22683-bib-0011] Bar M , Aminoff E , Schacter DL. 2008 Scenes unseen: The parahippocampal cortex intrinsically subserves contextual associations, not scenes or places per se. J Neurosci 28:8539–8544. 18716212 10.1523/JNEUROSCI.0987-08.2008 PMC2707255

[hipo22683-bib-0012] Bird CM , Shallice T , Cipolotti L. 2007 Fractionation of memory in medial temporal lobe amnesia. Neuropsychologia 45:1160–1171. 17129591 10.1016/j.neuropsychologia.2006.10.011

[hipo22683-bib-0013] Brandt KR , Eysenck MW , Nielsen MK , von Oertzen TJ. 2016 Selective lesion to the entorhinal cortex leads to an impairment in familiarity but not recollection. Brain Cogn 104:82–92. 26974041 10.1016/j.bandc.2016.02.005

[hipo22683-bib-0014] Brodeur MB , Dionne‐Dostie E , Montreuil T , Lepage M. 2010 The Bank of Standardized Stimuli (BOSS), a new set of 480 normative photos of objects to be used as visual stimuli in cognitive research. PLoS One 5:e10773‐ 20532245 10.1371/journal.pone.0010773 PMC2879426

[hipo22683-bib-0015] Büchel C , Holmes AP , Rees G , Friston KJ. 1998 Characterizing stimulus‐response functions using nonlinear regressors in parametric fMRI experiments. Neuroimage 8:140–148. 9740757 10.1006/nimg.1998.0351

[hipo22683-bib-0016] Buffalo EA , Bellgowan PSF , Martin A. 2006 Distinct roles for medial temporal lobe structures in memory for objects and their locations. Learn Mem 13:638–643. 16980544 10.1101/lm.251906 PMC1783618

[hipo22683-bib-0017] Burton AM , White D , McNeill A. 2010 The Glasgow face matching test. Behav Res Methods 42:286–291. 20160307 10.3758/BRM.42.1.286

[hipo22683-bib-0018] Cohn M , Moscovitch M , Lahat A , McAndrews MP. 2009 Recollection versus strength as the primary determinant of hippocampal engagement at retrieval. Proc Natl Acad Sci U S A 106:22451–22455. 20007783 10.1073/pnas.0908651106 PMC2799749

[hipo22683-bib-0019] Davachi L. 2006 Item, context and relational episodic encoding in humans. Curr Opin Neurobiol 16:693–700. 17097284 10.1016/j.conb.2006.10.012

[hipo22683-bib-0020] Devlin JT , Price CJ. 2007 Perirhinal contributions to human visual perception. Curr Biol 17:1484–1488. 17764947 10.1016/j.cub.2007.07.066 PMC1971135

[hipo22683-bib-0021] Diana RA , Yonelinas AP , Ranganath C. 2007 Imaging recollection and familiarity in the medial temporal lobe: A three‐component model. Trends Cogn Sci 11:379–386. 17707683 10.1016/j.tics.2007.08.001

[hipo22683-bib-0022] Diana RA , Yonelinas AP , Ranganath C. 2008 High‐resolution multi‐voxel pattern analysis of category selectivity in the medial temporal lobes. Hippocampus 18:536–541. 18446830 10.1002/hipo.20433 PMC2398650

[hipo22683-bib-0023] Dolcos F , LaBar KS , Cabeza R. 2005 Remembering one year later: Role of the amygdala and the medial temporal lobe memory system in retrieving emotional memories. Proc Natl Acad Sci U S A 102:2626–2631. 15703295 10.1073/pnas.0409848102 PMC548968

[hipo22683-bib-0024] Duarte A , Henson RN , Graham KS. 2011 Stimulus content and the neural correlates of source memory. Brain Res 1373:110–123. 21145314 10.1016/j.brainres.2010.11.086 PMC3098368

[hipo22683-bib-0025] Dudukovic NM , Preston AR , Archie JJ , Glover GH , Wagner AD. 2011 High‐resolution fMRI reveals match enhancement and attentional modulation in the human medial temporal lobe. J Cogn Neurosci 23:670–682. 20433244 10.1162/jocn.2010.21509 PMC5746189

[hipo22683-bib-0026] Eichenbaum H , Yonelinas AP , Ranganath C. 2007 The medial temporal lobe and recognition memory. Annu Rev Neurosci 30:123–152. 17417939 10.1146/annurev.neuro.30.051606.094328 PMC2064941

[hipo22683-bib-0027] Epstein R , Harris A , Stanley D , Kanwisher N. 1999 The Parahippocampal Place Area. Neuron 23:115–125. 10402198 10.1016/s0896-6273(00)80758-8

[hipo22683-bib-0028] Farovik A , Place RJ , Miller DR , Eichenbaum H. 2011 Amygdala lesions selectively impair familiarity in recognition memory. Nat Neurosci 14:1416–1417. 21946327 10.1038/nn.2919 PMC3203336

[hipo22683-bib-0029] Friston KJ , Fletcher P , Josephs O , Holmes A , Rugg MD , Turner R. 1998 Event‐related fMRI: Characterizing differential responses. Neuroimage 7:30–40. 9500830 10.1006/nimg.1997.0306

[hipo22683-bib-0030] Friston KJ , Penny WD , Glaser DE. 2005 Conjunction revisited. Neuroimage 25:661–667. 15808967 10.1016/j.neuroimage.2005.01.013

[hipo22683-bib-0031] Hannula DE , Libby LA , Yonelinas AP , Ranganath C. 2013 Medial temporal lobe contributions to cued retrieval of items and contexts. Neuropsychologia 51:2322–2332. 23466350 10.1016/j.neuropsychologia.2013.02.011 PMC3737273

[hipo22683-bib-0032] Von Der Heide RJ , Skipper LM , Olson IR. 2013 Anterior temporal face patches: A meta‐analysis and empirical study. Front Hum Neurosci 7:17– 23378834 10.3389/fnhum.2013.00017 PMC3561664

[hipo22683-bib-0033] Insausti R , Juottonen K , Soininen H , Insausti A , Partanen K , Vainio P , Laakso M , Pitkanen A. 1998 MR volumetric analysis of the human entorhinal, perirhinal, and temporopolar cortices. AJNR Am J Neuroradiol 19:659–671. 9576651 PMC8337393

[hipo22683-bib-0034] Jacoby LL. 1991 A process dissociation framework: Separating automatic from intentional uses of memory. J Mem Lang 30:513–541.

[hipo22683-bib-0035] Kafkas A , Montaldi D. 2012 Familiarity and recollection produce distinct eye movement, pupil and medial temporal lobe responses when memory strength is matched. Neuropsychologia 50:3080–3093. 22902538 10.1016/j.neuropsychologia.2012.08.001

[hipo22683-bib-0036] Kafkas A , Montaldi D. 2014 Two separate, but interacting, neural systems for familiarity and novelty detection: A dual‐route mechanism. Hippocampus 24:516–527. 24436072 10.1002/hipo.22241

[hipo22683-bib-0037] Kafkas A , Montaldi D. 2015a Striatal and midbrain connectivity with the hippocampus selectively boosts memory for contextual novelty. Hippocampus 25:1262–1273. 25708843 10.1002/hipo.22434 PMC4672698

[hipo22683-bib-0038] Kafkas A , Montaldi D. 2015b The pupillary response discriminates between subjective and objective familiarity and novelty. Psychophysiology 52:1305–1316. 26174940 10.1111/psyp.12471 PMC4737255

[hipo22683-bib-0039] Kensinger EA , Schacter DL. 2006 Amygdala activity is associated with the successful encoding of item, but not source, information for positive and negative stimuli. J Neurosci 26:2564–2570. 16510734 10.1523/JNEUROSCI.5241-05.2006 PMC6793660

[hipo22683-bib-0040] Kim J , Yassa MA. 2013 Assessing recollection and familiarity of similar lures in a behavioral pattern separation task. Hippocampus 23:287–294. 23401187 10.1002/hipo.22087 PMC4172605

[hipo22683-bib-0041] Konkel A , Cohen NJ. 2009 Relational memory and the hippocampus: Representations and methods. Front Neurosci 3:166–174. 20011138 10.3389/neuro.01.023.2009 PMC2751650

[hipo22683-bib-0042] LaBar KS , Cabeza R. 2006 Cognitive neuroscience of emotional memory. Nat Rev Neurosci 7:54–64. 16371950 10.1038/nrn1825

[hipo22683-bib-0043] Lee ACH , Buckley MJ , Pegman SJ , Spiers H , Scahill VL , Gaffan D , Bussey TJ , Davies RR , Kapur N , Hodges JR , Graham KS. 2005 Specialization in the medial temporal lobe for processing of objects and scenes. Hippocampus 15:782–797. 16010661 10.1002/hipo.20101

[hipo22683-bib-0044] Lee ACH , Scahill VL , Graham KS. 2008 Activating the medial temporal lobe during oddity judgment for faces and scenes. Cereb Cortex 18:683–696. 17615247 10.1093/cercor/bhm104

[hipo22683-bib-0045] Liang JC , Wagner AD , Preston AR. 2012 Content representation in the human medial temporal lobe. Cereb Cortex 23:80–96. 22275474 10.1093/cercor/bhr379 PMC3513952

[hipo22683-bib-0046] Litman L , Awipi T , Davachi L. 2009 Category‐specificity in the human medial temporal lobe cortex. Hippocampus 19:308–319. 18988234 10.1002/hipo.20515 PMC2649983

[hipo22683-bib-0047] Maldjian JA , Laurienti PJ , Kraft RA , Burdette JH. 2003 An automated method for neuroanatomic and cytoarchitectonic atlas‐based interrogation of fMRI data sets. Neuroimage 19:1233–1239. 12880848 10.1016/s1053-8119(03)00169-1

[hipo22683-bib-0048] Maldjian JA , Laurienti PJ , Burdette JH. 2004 Precentral gyrus discrepancy in electronic versions of the Talairach atlas. Neuroimage 21:450–455. 14741682 10.1016/j.neuroimage.2003.09.032

[hipo22683-bib-0049] Mandler G. 1980 Recognizing: The judgment of previous occurrence. Psychol Rev 87:252–271.

[hipo22683-bib-0050] Martin CB , Bowles B , Mirsattari SM , Köhler S. 2011 Selective familiarity deficits after left anterior temporal‐lobe removal with hippocampal sparing are material specific. Neuropsychologia 49:1870–1878. 21419788 10.1016/j.neuropsychologia.2011.03.012

[hipo22683-bib-0051] Martin CB , McLean DA , O'Neil EB , Köhler S. 2013 Distinct Familiarity‐Based Response Patterns for Faces and Buildings in Perirhinal and Parahippocampal Cortex. J Neurosci 33:10915–10923. 23804111 10.1523/JNEUROSCI.0126-13.2013 PMC6618503

[hipo22683-bib-0052] Martin CB , Cowell RA , Gribble PL , Wright J , Köhler S. 2016 Distributed category‐specific recognition‐memory signals in human perirhinal cortex. Hippocampus 26:423–436. 26385759 10.1002/hipo.22531

[hipo22683-bib-0053] Mattavelli G , Sormaz M , Flack T , Asghar AUR , Fan S , Frey J , Manssuer L , Usten D , Young AW , Andrews TJ. 2014 Neural responses to facial expressions support the role of the amygdala in processing threat. Soc Cogn Affect Neurosci 9:1684–1689. 24097376 10.1093/scan/nst162 PMC4221207

[hipo22683-bib-0054] Mayes AR , Holdstock JS , Isaac CL , Hunkin NM , Roberts N. 2002 Relative sparing of item recognition memory in a patient with adult‐onset damage limited to the hippocampus. Hippocampus 12:325–340. 12099484 10.1002/hipo.1111

[hipo22683-bib-0055] Mayes AR , Montaldi D , Migo E. 2007 Associative memory and the medial temporal lobes. Trends Cogn Sci 11:126–135. 17270487 10.1016/j.tics.2006.12.003

[hipo22683-bib-0056] Migo EM , Mayes AR , Montaldi D. 2012 Measuring recollection and familiarity: Improving the remember/know procedure. Conscious Cogn 21:1435–1455. 22846231 10.1016/j.concog.2012.04.014

[hipo22683-bib-0057] Montaldi D , Mayes AR. 2010 The role of recollection and familiarity in the functional differentiation of the medial temporal lobes. Hippocampus 20:1291–1314. 20928828 10.1002/hipo.20853

[hipo22683-bib-0058] Montaldi D , Spencer TJ , Roberts N , Mayes AR. 2006 The neural system that mediates familiarity memory. Hippocampus 16:504–520. 16634088 10.1002/hipo.20178

[hipo22683-bib-0059] Mundy ME , Downing PE , Dwyer DM , Honey RC , Graham KS. 2013 A Critical role for the hippocampus and perirhinal Cortex in perceptual learning of scenes and faces: Complementary findings from amnesia and fMRI. J Neurosci 33:10490–10502. 23785161 10.1523/JNEUROSCI.2958-12.2013 PMC3722491

[hipo22683-bib-0060] Nasr S , Tootell RBH. 2012 Role of fusiform and anterior temporal cortical areas in facial recognition. Neuroimage 63:1743–1753. 23034518 10.1016/j.neuroimage.2012.08.031 PMC3472036

[hipo22683-bib-0061] Nestor A , Plaut DC , Behrmann M. 2011 Unraveling the distributed neural code of facial identity through spatiotemporal pattern analysis. Proc Natl Acad Sci U S A 108:9998–10003. 21628569 10.1073/pnas.1102433108 PMC3116398

[hipo22683-bib-0062] Norman KA , Polyn SM , Detre GJ , Haxby JV. 2006 Beyond mind‐reading: Multi‐voxel pattern analysis of fMRI data. Trends Cogn Sci 10:424–430. 16899397 10.1016/j.tics.2006.07.005

[hipo22683-bib-0063] O'Neil EB , Cate AD , Köhler S. 2009 Perirhinal cortex contributes to accuracy in recognition memory and perceptual discriminations. J Neurosci 29:8329–8334. 19571124 10.1523/JNEUROSCI.0374-09.2009 PMC6665676

[hipo22683-bib-0064] Olson IR , Ezzyat Y , Plotzker A , Chatterjee A. 2015 The end point of the ventral visual stream: Face and non‐face perceptual deficits following unilateral anterior temporal lobe damage. Neurocase 21:554–562. 25238048 10.1080/13554794.2014.959025 PMC4366355

[hipo22683-bib-0065] Pitkänen A. 2000 Connectivity of the rat amygdaloid complex In: Aggleton J, editor. The Functional Analysis of the Amygdala. New York: Wiley‐Liss pp 31–115.

[hipo22683-bib-0066] Pitkänen A , Kemppainen S. 2002 Comparison of the distribution of calcium‐binding proteins and intrinsic connectivity in the lateral nucleus of the rat, monkey, and human amygdala. Pharmacol Biochem Behav 71:369–377. 11830171 10.1016/s0091-3057(01)00690-6

[hipo22683-bib-0067] Preston AR , Bornstein AM , Hutchinson JB , Gaare ME , Glover GH , Wagner AD. 2010 High‐resolution fMRI of content‐sensitive subsequent memory responses in human medial temporal lobe. J Cogn Neurosci 22:156–173. 19199423 10.1162/jocn.2009.21195 PMC2854293

[hipo22683-bib-0068] Ranganath C , Yonelinas AP , Cohen MX , Dy CJ , Tom SM , D'Esposito M. 2004 Dissociable correlates of recollection and familiarity within the medial temporal lobes. Neuropsychologia 42:2–13. 14615072 10.1016/j.neuropsychologia.2003.07.006

[hipo22683-bib-0069] Rasmussen CE , Williams CK. 2006 Gaussian processes for machine learning. Cambridge, MA: MIT Press.

[hipo22683-bib-0070] Rossion B , Hanseeuw B , Dricot L. 2012 Defining face perception areas in the human brain: A large‐scale factorial fMRI face localizer analysis. Brain Cogn 79:138–157. 22330606 10.1016/j.bandc.2012.01.001

[hipo22683-bib-0071] Rugg MD , Vilberg KL. 2013 Brain networks underlying episodic memory retrieval. Curr Opin Neurobiol 23:255–260. 23206590 10.1016/j.conb.2012.11.005 PMC3594562

[hipo22683-bib-0072] Rugg MD , Vilberg KL , Mattson JT , Yu SS , Johnson JD , Suzuki M. 2012 Item memory, context memory and the hippocampus: fMRI evidence. Neuropsychologia 50:3070–3079. 22732490 10.1016/j.neuropsychologia.2012.06.004 PMC3472091

[hipo22683-bib-0073] Schrouff J , Rosa MJ , Rondina JM , Marquand AF , Chu C , Ashburner J , Phillips C , Richiardi J , Mourão‐Miranda J. 2013 PRoNTo: Pattern recognition for neuroimaging toolbox. Neuroinformatics 11:319–337. 23417655 10.1007/s12021-013-9178-1 PMC3722452

[hipo22683-bib-0074] Schwartz CE , Wright CI , Shin LM , Kagan J , Whalen PJ , McMullin KG , Rauch SL. 2003 Differential amygdalar response to novel versus newly familiar neutral faces: A functional MRI probe developed for studying inhibited temperament. Biol Psychiatry 53:854–862. 12742672 10.1016/s0006-3223(02)01906-6

[hipo22683-bib-0075] Skinner EI , Fernandes MA. 2007 Neural correlates of recollection and familiarity: A review of neuroimaging and patient data. Neuropsychologia 45:2163–2179. 17445844 10.1016/j.neuropsychologia.2007.03.007

[hipo22683-bib-0076] Smith CN , Wixted JT , Squire LR. 2011 The hippocampus supports both recollection and familiarity when memories are strong. J Neurosci 31:15693–15702. 22049412 10.1523/JNEUROSCI.3438-11.2011 PMC3220416

[hipo22683-bib-0077] Squire LR , Wixted JT , Clark RE. 2007 Recognition memory and the medial temporal lobe: A new perspective. Nat Rev Neurosci 8:872–883. 17948032 10.1038/nrn2154 PMC2323975

[hipo22683-bib-0078] Staresina BP , Duncan KD , Davachi L. 2011 Perirhinal and parahippocampal cortices differentially contribute to later recollection of object‐ and scene‐related event details. J Neurosci 31:8739–8747. 21677158 10.1523/JNEUROSCI.4978-10.2011 PMC3128497

[hipo22683-bib-0079] Staresina BP , Cooper E , Henson RN. 2013 Reversible information flow across the medial temporal lobe: The hippocampus links cortical modules during memory retrieval. J Neurosci 33:14184–14192. 23986252 10.1523/JNEUROSCI.1987-13.2013 PMC3756762

[hipo22683-bib-0080] Suzuki WA , Amaral DG. 1994 Perirhinal and parahippocampal cortices of the macaque monkey: Cortical afferents. J Comp Neurol 350:497–533. 7890828 10.1002/cne.903500402

[hipo22683-bib-0081] Taylor KJ , Henson RNA , Graham KS. 2007 Recognition memory for faces and scenes in amnesia: Dissociable roles of medial temporal lobe structures. Neuropsychologia 45:2428–2438. 17509626 10.1016/j.neuropsychologia.2007.04.004

[hipo22683-bib-0082] de Vanssay‐Maigne A , Noulhiane M , Devauchelle AD , Rodrigo S , Baudoin‐Chial S , Meder JF , Oppenheim C , Chiron C , Chassoux F. 2011 Modulation of encoding and retrieval by recollection and familiarity: Mapping the medial temporal lobe networks. Neuroimage 58:1131–1138. 21763430 10.1016/j.neuroimage.2011.06.086

[hipo22683-bib-0083] Watson HC , Wilding EL , Graham KS. 2012 A role for perirhinal cortex in memory for novel object‐context associations. J Neurosci 32:4473–4481. 22457495 10.1523/JNEUROSCI.5751-11.2012 PMC6622046

[hipo22683-bib-0084] Wixted JT , Mickes L , Squire LR. 2010 Measuring recollection and familiarity in the medial temporal lobe. Hippocampus 20:1195–1205. 20848603 10.1002/hipo.20854 PMC2975576

[hipo22683-bib-0085] Wright CI , Martis B , Schwartz CE , Shin LM , Fischer H. å , McMullin K , Rauch SL. 2003 Novelty responses and differential effects of order in the amygdala, substantia innominata, and inferior temporal cortex. Neuroimage 18:660–669. 12667843 10.1016/s1053-8119(02)00037-x

[hipo22683-bib-0086] Yonelinas AP , Otten LJ , Shaw KN , Rugg MD. 2005 Separating the brain regions involved in recollection and familiarity in recognition memory. J Neurosci 25:3002–3008. 15772360 10.1523/JNEUROSCI.5295-04.2005 PMC6725129

[hipo22683-bib-0087] Yonelinas AP. 2002 The nature of recollection and familiarity: A review of 30 years of research. J Mem Lang 46:441–517.

[hipo22683-bib-0088] Yonelinas AP , Widaman K , Mungas D , Reed B , Weiner MW , Chui HC. 2007 Memory in the aging brain: Doubly dissociating the contribution of the hippocampus and entorhinal cortex. Hippocampus 17:1134–1140. 17636547 10.1002/hipo.20341 PMC2194291

[hipo22683-bib-0089] Young AW , Aggleton JP , Hellawell DJ , Johnson M , Broks P , Hanley JR. 1995 Face processing impairments after amygdalotomy. Brain 118:15–24. 7895001 10.1093/brain/118.1.15

[hipo22683-bib-0090] Zhuo J , Fan L , Liu Y , Zhang Y , Yu C , Jiang T. 2016 Connectivity profiles reveal a transition subarea in the parahippocampal region that integrates the anterior temporal‐posterior medial systems. J Neurosci 36:2782–2795. 26937015 10.1523/JNEUROSCI.1975-15.2016 PMC6604873

