# Supplementary material for: Material Specificity Drives Medial Temporal Lobe Familiarity But Not Hippocampal Recollection
Source: Hippocampus. 2016 Dec 26;27(2):194–209. doi: 10.1002/hipo.22683 (PMC5299537; doi:10.1002/hipo.22683)
Supplement: Supplementary file 1 — Supporting Information [file HIPO-27-194-s001.docx]

**Supplementary Table 1.** Mean number of trials (SD in parenthesis) for the 4 response categories used in the parametric analyses for the three types of stimuli

| **Stimulus type** | **Response** | | | |
| --- | --- | --- | --- | --- |
|  | **M** | **F1** | **F2** | **F3** |
| Scenes | 22.56 (10.05) | 21.17 (12.5) | 16.00 (6.8) | 19.56 (11.13) |
| Objects | 18.43 (10.03) | 12.00 (2.90) | 18.33 (9.94) | 32.72 (15.58) |
| Faces | 32.78 (13.05) | 21.11 (11.13) | 10.17 (9.71) | 22.28 (9.36) |
| Total | 73.77 (39.94) | 54.28 (27.91) | 44.5 (21.22) | 74.56 (35.57) |

**Supplementary Table 2**

Alternative classification analysis with multiple binary SVM (Support Vector Machine) classifications for all combinations (i.e., scenes vs objects; scenes versus faces; objects versus faces)

|  | **Scenes** | **Objects** | **Faces** |
| --- | --- | --- | --- |
| **ROIs** | **Accuracy %** | **Accuracy %** | **Accuracy %** |
| **Whole-brain** | **68.8*** | **84.4**** | **68.75**** |
| **HC** | 56.25 | 62.5 | 56.25 |
| **Left HC** | 56.3 | 62.5 | 56.3 |
| **Right HC** | 59.4 | 56.3 | 53.15 |
| **PRC** | 50 | **75*** | 37.55 |
| **Left PRC** | 65.65 | 62.5 | 37.5 |
| **Right PRC** | 59.4 | **78.15**** | 34.4 |
| **ERC** | 59.4 | **78.15*** | 37.5 |
| **Left ERC** | 46.9 | 56.3 | 50.05 |
| **Right ERC** | 65.65 | **75*** | 40.65 |
| **PHC** | **65.65** | **78.15‡** | 59.4 |
| **Left PHC** | **75.65*** | 68.75 | 56.25 |
| **Right PHC** | **72.5*** | **71.9*** | 46.9 |
| **AMG** | 37.55 | 59.4 | 56.25 |
| **Left AMG** | 46.9 | 59.4 | **64.65⊥** |
| **Right AMG** | 37.55 | 52.9 | **75*** |

*Note:* The classification accuracy (%) is the mean accuracy for each stimulus type across all combinations; * *P* < 0.05; ** *P* < 0.01; ‡ *P* = 0.06 (trend); **⊥** *P* = 0.08 (trend)**;** *P*-values are calculated from permutation testing with 1000 permutations.

**Supplementary Table 3.** Classification accuracy (%) at the group level for F3 responses across the three types of stimuli for the whole brain and the regions of interest (ROIs)

|  | **Scenes** | **Objects** | **Faces** |
| --- | --- | --- | --- |
| **ROIs** | **Accuracy %** | **Accuracy %** | **Accuracy %** |
| **Whole-brain** | **56.3^*^** | **68.8*** | **56.3*** |
| **HC** | 47.1 | 44.4 | 56.3 |
| **Left HC** | 45.5 | 42.9 | 56.3 |
| **Right HC** | 50 | 37.5 | 31.3 |
| **PRC** | 12.5 | **75**** | 31.3 |
| **Left PRC** | 0.0 | **81.3**** | 43.8 |
| **Right PRC** | 50 | **81.3**** | 6.3 |
| **ERC** | 37.5 | **75*** | 31.25 |
| **Left ERC** | 12.5 | **75**** | 50 |
| **Right ERC** | 50 | **75**** | 31.25 |
| **PHC** | **68.8*** | 68.8 | 25 |
| **Left PHC** | **75*** | 62.5 | 25 |
| **Right PHC** | 68.8 | **75**** | 18.8 |
| **AMG** | 6.3 | 50 | 56.3 |
| **Left AMG** | 12.5 | 56.3 | **56.3*** |
| **Right AMG** | 25 | 50 | **62.5*** |

*Note:* * *P* < 0.05; ** *P* < 0.01; *P*-values are calculated from permutation testing with 1000 permutations.

**Supplementary Table 4.** Classification accuracy (%) at the group level for F3 and R responses, collapsed for the three types of stimuli, within the whole brain and the regions of interest (ROIs)

| **ROIs** | **F3 Accuracy (%)** | **R Accuracy (%)** |
| --- | --- | --- |
| **Whole-brain** | **75*** | **100***** |
| **HC** | 37.5 | **75*** |
| **Left HC** | 43.8 | **81.3*** |
| **Right HC** | 31.3 | **87.5***** |
| **PRC** | **75*** | 68.5 |
| **Left PRC** | 56.3 | **81.3*** |
| **Right PRC** | 68.8 | 56.3 |
| **ERC** | 62.5 | **84.4***** |
| **Left ERC** | 43.8 | 68.8 |
| **Right ERC** | 50 | 56.3 |
| **PHC** | 62.5 | **87.5**** |
| **Left PHC** | **81.3**** | **81.3*** |
| **Right PHC** | 56.3 | 62.5 |
| **AMG** | 62.5 | 62.5 |
| **Left AMG** | 43.8 | 56.3 |
| **Right AMG** | 50 | 50 |

*Note:* * *P* < 0.05; ** *P* < 0.01; *** *P* < 0.001; P-values are calculated from permutation testing with 1000 permutations.

**Supplementary Table 5.** Classification accuracy (%) at the group level for F3 responses relative to misses for scenes, objects and faces, within the whole brain and the regions of interest (ROIs)

|  | **Scenes** | **Objects** | **Faces** |
| --- | --- | --- | --- |
| **ROIs** | **Accuracy %** | **Accuracy %** | **Accuracy %** |
| **Whole-brain** | **65.6*** | **82.3*** | **75**** |
| **HC** | 44 | 52.9 | 43.7 |
| **Left HC** | 62.5 | 52.9 | 56.25 |
| **Right HC** | 50 | 47 | 50 |
| **PRC** | 38.9 | 64.7 | 56.2 |
| **Left PRC** | 43.8 | **65.6‡** | 50 |
| **Right PRC** | 50 | **76.5*** | 59.4 |
| **ERC** | 56.2 | 47 | 50 |
| **Left ERC** | 56.2 | 56.3 | **68.75*** |
| **Right ERC** | 62.5 | **68.7‡** | 31.25 |
| **PHC** | **75*** | **70**** | 31.25 |
| **Left PHC** | **68.8*** | **88**** | 68.75 |
| **Right PHC** | 50 | **71*** | 50 |
| **AMG** | 43.7 | 58.8 | **65.6‡** |
| **Left AMG** | 62.5 | 52.9 | **75*** |
| **Right AMG** | 56.2 | 58.8 | 62.5 |

*Note:* * *P* < 0.05; ** *P* < 0.01; ‡ *P* = 0.06 (trend); *P*-values are calculated from permutation testing with 1000 permutations.
